# Integration of light quality signals regulates ABA abundance and stomatal movements during seedling establishment

**DOI:** 10.1111/nph.70746

**Published:** 2025-11-23

**Authors:** Mathilda Gustavsson, Lionel Hill, Keara A. Franklin, Ashley J. Pridgeon

**Affiliations:** ^1^ School of Biological Sciences, Life Sciences Building University of Bristol Bristol BS8 1TQ UK; ^2^ John Innes Centre Norwich Research Park Norwich NR4 7UH UK

**Keywords:** abscisic acid, light signalling, seedling establishment, seedling establishment, shade avoidance, signal integration, stomata, UV‐B signalling

## Abstract

- Obtaining sufficient light for photosynthesis and avoiding desiccation are two key challenges faced by seedlings during early establishment. Perception of light quality via specialised photoreceptors signals the availability of sunlight for photosynthesis. Canopy shade is depleted in red (R) and enriched in far‐red (FR) light, lowering R : FR ratio, while direct sunlight and sunflecks contain UV‐B. The balance between these wavelengths can determine the developmental strategy adopted by seedlings to either avoid shade, via stem elongation, or promote the expansion of photosynthetic organs. How seedlings regulate stomatal movements in different light environments is poorly understood.
- Using FR and UV‐B supplementation to mimic aspects of canopy shade and sunlight, respectively, we monitored stomatal apertures in *Arabidopsis thaliana* cotyledons and gas exchange in the cotyledons of Chinese kale (*Brassica oleracea* var alboglabra).
- We show that low R : FR inhibits stomatal opening via a mechanism involving PHYTOCHROME INTERACTING FACTOR 4 (PIF4) and increased abscisic acid (ABA). UV‐B perceived by the UV RESISTANCE LOCUS 8 (UVR8) photoreceptor acts antagonistically, promoting stomatal opening in a response that requires phototropin photoreceptors.
- The convergence of phytochrome and UVR8 signalling to control ABA abundance enables plants to coordinate stem elongation and water use, potentially facilitating seedling establishment in dynamic light environments.

## Introduction

Plants have evolved a suite of photoreceptors to detect light quality and quantity, signalling the availability of sunlight to fuel photosynthesis. Seedlings emerging under canopy shade are exposed to increased amounts of FR light, reflected and transmitted from vegetative tissue. This lowers the ratio of red to far‐red light (R : FR), inactivating phytochrome photoreceptors and driving stem elongation to overtop competitors (Ballare *et al*., 1990). Direct sunlight and sunflecks contain UV‐B light, which antagonises shade avoidance responses (Hayes *et al*., 2014; Sharma *et al*., 2019; Tavridou *et al*., 2020a), promoting a compact stature (Moriconi *et al*., 2018). Continuous monitoring of light quality changes, therefore, enables plants to optimise developmental strategy in fluctuating environments (Fernández‐Milmanda & Ballaré, 2021).

Mortality rates are high during early seedling establishment (Leck *et al*., 2008), with water scarcity presenting a significant stress (Fenner & Thompson, 2005). Stomata are microscopic pores found predominantly on the epidermis of leaves. They perform a crucial role in the prevention of water loss and the uptake of carbon through the regulation of gas exchange. This can involve short‐term alterations in the size of the stomatal pore and longer term alterations in stomatal development, changing the number of stomatal pores on a plant's surface (Lawson & Matthews, 2020). Stomata are sensitive to a range of exogenous and endogenous signals (including light, temperature, CO_2_ concentration, water availability, pathogen and microbe‐associated signals). The signalling processes involved in blue light‐induced stomatal opening (Inoue & Kinoshita, 2017) and abscisic acid (ABA)‐induced stomatal closure are well characterised (Munemasa *et al*., 2015; Lawson & Matthews, 2020). Although guard cells can respond autonomously to environmental signals such as blue light and ABA, responses can be modulated by mesophyll‐produced signals such as sugar and malate to coordinate stomatal aperture with leaf carbon assimilation (Lawson & Matthews, 2020; Flütsch & Santelia, 2021). Red light‐mediated stomatal opening is less well characterised than the corresponding blue light response. As the red‐light response saturates at similar light intensities to photosynthesis, it is thought to be linked to photosynthetic carbon assimilation; however, the exact nature of this link is unclear (Matthews *et al*., 2020; Taylor *et al*., 2024). The red‐light photoreceptor, phytochrome B (phyB) and downstream phytochrome signalling components have been shown to contribute to the coordination of stomatal apertures (Wang *et al*., 2010; Li *et al*., 2022; Rovira *et al*., 2024).

In this study, we analysed how light quality signals controlling light foraging affect stomatal aperture in developing seedlings. Stomatal development is established during embryogenesis, with stomata forming rapidly after germination (Smit *et al*., 2023). The stomata of young seedlings are responsive to light signals (Rovira *et al*., 2024), but the role of stomatal movement during seedling establishment is poorly understood. Stomatal apertures were quantified from cotyledons treated with white light (WL) supplemented with FR and/or low‐dose UV‐B to simulate aspects of canopy shade and direct sunlight, respectively. We show that low R : FR inhibits stomatal opening in cotyledons in a response involving the basic helix–loop–helix (bHLH) transcription factor, PIF4 and accumulation of ABA. By contrast, UV‐B supplementation promotes the sustained opening of stomata in a process requiring the UVR8 and phototropin photoreceptors as well as the signalling component BLUE LIGHT SIGNALLING 1 (BLUS1). When low R : FR and UV‐B signals are combined, UV‐B signalling overrides the effects of low R : FR on ABA abundance and stomatal aperture. Our data suggest that the integration of light quality signals by multiple photoreceptors coordinates seedling light‐foraging strategy with water use through the alteration of hormone levels.

## Materials and Methods

### Plant material and growth conditions

*Arabidopsis thaliana* seedlings and Chinese kale (*Brassica oleracea* var Alboglabra) were grown in a 3 : 1 Levingtons F2 compost: silver sand (Melcourt) mixture. Seeds were washed with 70% ethanol, sown on soil and stratified for 2–3 d in the dark at 4°C. Seedlings were grown in long‐day conditions (16 h : 8 h, day : night) in 75 μmol m^−2^ s^−1^ WL (see Supporting Information Fig. S1 for light spectra) at a constant humidity of 70% and a temperature of 20°C in growth cabinets (Microclima 1600E; Snijder Scientific, Tilburg, the Netherlands). All Arabidopsis genotypes are in a Col‐0 background apart from the *hy5hyh*, and *phyB* mutants, which are in the Wassilewskija (Ws) background. A list of genotypes (McNellis *et al*., 1994; Shirley *et al*., 1995; Kagawa *et al*., 2001; Holm *et al*., 2002; Yoshida *et al*., 2002; Franklin *et al*., 2003a; Lee *et al*., 2006; de Lucas *et al*., 2008; Leivar *et al*., 2008a,b; Favory *et al*., 2009; Park *et al*., 2009; Frey *et al*., 2012) and their descriptions can be found in Table S1.

### Light treatments

**Fig. 1:**
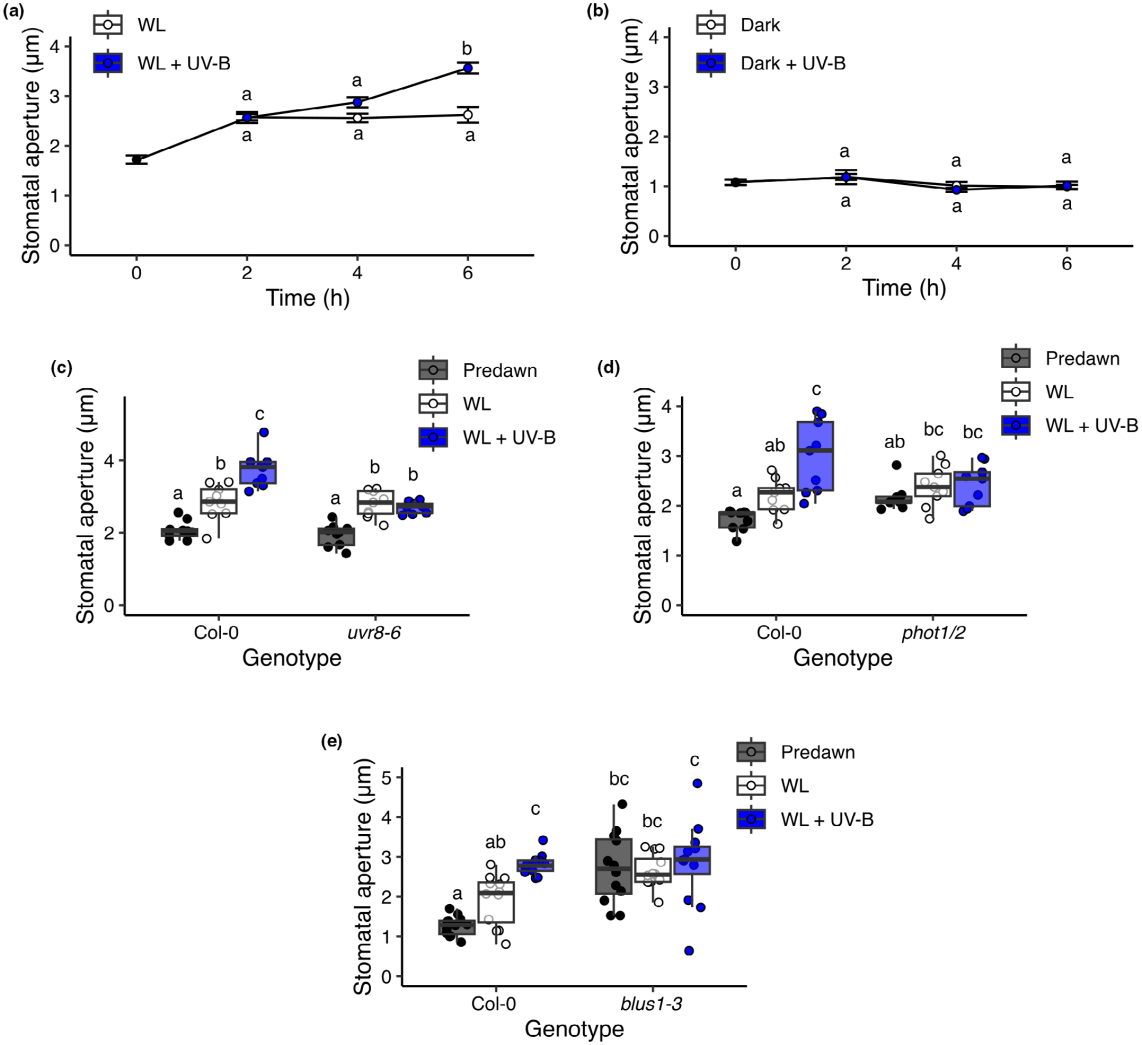
Low‐dose UV‐B enhances stomatal opening in a UVR8‐ and phototropin‐dependent manner. Stomatal apertures of 7‐d‐old Arabidopsis seedlings in response to different light treatments. Seedlings were treated with (a) white light (WL) ± UV‐B and (b) darkness ± UV‐B (Dark + UV‐B) over a 6‐h time course. Data are presented as the mean ± SE of each plant's average stomatal aperture. The stomatal responses of (c) *uvr8‐6*, (d) *phot1/2*, and (e) *blus1‐3* mutants following 6 h of WL ± UV‐B treatment. Treatments were started at dawn, and apertures were measured before dawn (predawn). Data are presented as boxplots showing the median and interquartile range of each group. The upper and lower whiskers represent data within 1.5 × IQR. Each individual plant's mean stomatal aperture is represented as a point on the plot. For all genotype treatment combinations, *n* = 9–12 seedlings over three independent experiments. Each mean seedling stomatal aperture was calculated from 10 to 12 stomatal measurements. Data were analysed using a two‐way ANOVA, followed by Tukey multiple comparison test.

Light measurements were performed using an Ocean Optics FLAME‐S‐UV–VIS spectrometer with a cosine corrector. For UV‐B treatments, a Philips TL100W/01 narrowband tube light wrapped in strips of heatproof tape was used to provide treatments of 1 μmol m^−2^ s^−1^ UV‐B light (between 280 and 315 nm). Other than in Fig. 1(b), UV‐B was applied in a background of 75 μmol m^−2^ s^−1^ WL. For FR supplementation, LEDs emitting at a max peak of 735 nm were used in conjunction with 75 μmol m^−2^ s^−1^ WL. R : FR ratios were determined by dividing R light photon irradiance (between 660–670 nm) by FR light photon irradiance (between 725–735 nm). Low R : FR conditions were set between 0.06 and 0.08, whereas high R : FR conditions were between 4 and 7. During gas exchange measurements, light treatments were applied to the measured leaves using the red, blue and FR LEDs within the Licor 6800 multiphase flash fluorometer, while the plant outside the measuring cuvette was treated with light as described above.

### Stomatal aperture measurements

Arabidopsis seedlings were grown for 7 d in long‐day (16 h : 8 h, light : dark) conditions and transferred at dawn on the 8^th^ day to the appropriate light treatment, unless otherwise stated. Chinese kale seedlings were grown for 8 d and transferred at dawn on the 9^th^ day to appropriate light treatments. The length of light treatment is indicated in each figure. The aerial portion of the seedling was rapidly placed on a microscope slide, and images were taken of the abaxial surface of the cotyledons using a ×40 lens on a Zeiss Axiovert 200M inverted microscope fitted with a Hamamatsu ORCA‐ER digital camera. Images were randomised and stomatal apertures (pore widths) were then measured using Fiji (ImageJ) (Schindelin *et al*., 2012). For each experiment, stomatal apertures were measured predawn in addition to control and light treatments. Each experiment was repeated independently at least three times, with 3–4 plants measured per genotype per treatment. For Arabidopsis, a total of 10–12 stomatal apertures were measured per plant, and the average of these stomatal aperture values was considered *n* = 1. For Chinese kale, a total of 12–28 stomatal apertures were measured per plant, with the average of these values considered *n* = 1. Overall, each experiment has *n* = 9–12 for each genotype and treatment combination unless otherwise stated.

For Fig. S2(d,e), leaf discs and epidermal peels were harvested from the 5^th^ and 6^th^ rosette leaves of 4–5‐wk‐old plants grown in the same conditions as previously described for seedlings. Leaf discs were harvested using a 4‐mm biopsy punch. After tissue harvesting, leaf discs and epidermal peels were transferred to 50‐mm petri dishes containing 10 ml 10/50 buffer (10 mM MES, 50 mM KCl, pH adjusted to 6.15 using KOH) prewarmed to 20°C. Dishes were incubated in darkness for 2 h before predawn samples were removed for measurement. Remaining dishes were transferred to 75 μmol m^−2^ s^−1^ WL or WL supplemented with 1 μmol m^−2^ s^−1^ UV‐B. Leaf discs were measured 2, 4, and 6 h after treatment. Epidermal peels were measured 6 h after treatment. Each experiment was repeated independently at least three times, with three plants measured per genotype per treatment. A total of 10 stomatal apertures were measured per plant, and the average of these stomatal aperture values was considered *n* = 1. Overall, each experiment has *n* = 9 for each genotype and treatment combination.

### Gas exchange measurements

Chinese kale seedlings were grown for 14–18 d in long‐day conditions. On the day of measurement, seedlings were kept in darkness until the light treatment. Gas exchange measurements were recorded from seedling cotyledons using a Licor 6800 fitted with a multiphase flash fluorometer with conditions set to a constant flow: 400 μmol m^−2^ s^−1^, *T*_air_: 20°C, sample CO_2_: 400 ppm, sample H_2_O: 15 000 ppm, and mixing fan set to 7000 rpm. Cotyledon gas exchange was monitored in darkness for 30 min before treatment with 75 μmol m^−2^ s^−1^ red and blue light (67 and 8 μmol m^−2^ s^−1^, respectively) ± 10 μmol m^−2^ s^−1^ far‐red light for 2 h (see Fig. S1b for light spectra).

### Stomatal density measurements

Arabidopsis seedlings were grown for 7 d in long‐day (16 h : 8 h, light : dark) conditions. On the 8^th^ day, cotyledons were harvested and cleared (Sharma, 2017). Cleared tissue was imaged using a ×20 DIC lens on a Leica DMIRE2 microscope with a Leica DFC350 FX camera. A stack of images containing both the abaxial and adaxial epidermises was taken. Stomata were counted using the cell counter plugin of Fiji (ImageJ) within a 0.208‐mm^2^ region.

### ABA quantification

Arabidopsis seedlings were grown and treated similarly to stomatal aperture experiments. After light treatment, aerial seedling tissues were rapidly harvested, weighed and flash frozen in liquid nitrogen. Seedling tissue was ground into a fine powder and resuspended in 1.9 ml extraction buffer (1% v/v acetic acid in 100% isopropanol), spiked with 10 μl d6‐ABA (2.5 μg ml^−1^ in 100% MeOH). Samples were mixed overnight at 4°C and spun at 13.4 krcf for 5 min at 4°C. Supernatant was evaporated 950 μl at a time into a new tube using an Eppendorf Concentrator Plus set to V‐AL mode at 45°C for 1 h. The original sample tubes were resuspended in 950 μl of extraction buffer (without d6‐ABA) and centrifuged further for an hour at 4°C. The sample was centrifuged again at 13.4 krcf at 4°C, and the supernatant was transferred to the tube containing the evaporated sample, which was evaporated a final time using the same settings as discussed before. Dried samples were stored at −70°C until mass spectrometry analysis.

Dried samples were resuspended in 100 μl 100% MeOH and run on an Acquity UPLC equipped with a XevoTQS tandem mass spec (Waters, Milford, MA, USA). Separation was performed on a 50 × 2.1 mm 2.6 μ Kinetex EVO C18 column (Phenomenex, Torrance, CA, USA) using the following gradient of acetonitrile vs 0.1% formic acid in water, run at 0.7 ml min^−1^ and 30°C (0 min – 5%, 3 min – 95%, 3.5 min – 95%, 3.6 min – 5%, 5.1 min – 5%). Samples were maintained at 10°C, and the instrument injected 5 μl. Hormones were detected by negative mode electrospray, with the following mass transitions: ABA, 263 > 153; d6‐ABA, 269 > 159 (collision energy 10 V). Spray chamber conditions were 900 l h^−1^ drying gas at 500°C, 150 l h^−1^ cone gas, 7.0 bar nebuliser pressure and a spray voltage of 1.5 kV.

### Thermal imaging

Thermal images were recorded using a FLIR A665sc thermal imaging camera. ResearchIR software (v.4.40.9.30) was used to generate TIF images, which were then analysed using Fiji (ImageJ). Cotyledon temperatures were analysed within a 24‐pixel box, and the mean of the 24 pixels was used to represent each cotyledon's temperature.

### Chlorophyll fluorescence

Chlorophyll fluorescence parameters of 7‐d‐old seedlings were measured using an IMAGING‐PAM M series system with ImagingWin software (v.2.56p, Walz). Seedlings were treated with WL ± UV‐B and ± FR for 6 h. Following this, seedlings were dark‐adapted for 30 min before applying a saturating pulse of light. Maximal photosystem II efficiency (*F*_V_/*F*_M_) was calculated using the formula *F*_V_/*F*_M_ = (*F*_M_ – *F*_0_)/*F*_M_, where *F*_0_ and *F*_M_ represent fluorescence measurements before and after the saturating light pulse, respectively. Images were exported from the ImagingWin software as .tif files and analysed using Fiji (ImageJ). Manual thresholding was used to generate masks of the seedling aerial tissue before calculating the mean *F*_V_/*F*_M_ for that region of interest.

### RNAseq analysis

The raw count RNAseq data from GSE146125 (Tavridou *et al*., 2020b) and GSE192469 (Sharma *et al*., 2023) were downloaded from the NCBI GEO database. These studies treated 7‐d‐old Arabidopsis seedlings with supplemental far‐red and/or UV‐B for 3 and 4 h, respectively. EdgeR (v.4.6.2) was used to filter out low‐expressed genes, normalise sample library sizes and perform differential expression analysis using the quasi‐likelihood pipeline (quasi‐likelihood negative binomial model). Genes with FDRs (calculated using the Benjamini‐Hochberg method) less than 0.05 were classed as differentially regulated. Heat maps were generated for genes tagged with ABA biosynthesis, catabolism and signalling GO terms. Normalised log_2_(CPM) *Z*‐scores were used for heat map visualisation.

### qPCR analysis

Arabidopsis aerial tissue was harvested similarly to ABA quantification. RNA was extracted from samples using the Spectrum Total RNA kit (Sigma, St Louis, MO, USA). cDNA was synthesised using the high‐capacity cDNA reverse transcription kit with RNase inhibitor (Applied Biosystems, Foster City, CA, USA). qPCR was performed using the Brilliant III Ultra‐Fast SYBR Green qPCR Master Mix (Agilent, Santa Clara, CA, USA) and an Mx3000P (Agilent). Transcript abundances were calculated using the ∆∆*C*_t_ method, with *PP2A* and *ACT2* used as reference genes unless otherwise stated. Statistics were performed using the −∆∆*C*_t_ values. Primers are described in Table S2.

### Data presentation and analysis

All data were statistically analysed in R (v.4.3.1) (R Core Team, 2021) and plotted using the ggplot2 package (Wickham, 2016).

### Statistical analysis

Data were analysed using R (v.4.3.1). Datasets were analysed using one or two‐way ANOVAs with *post hoc* Tukey multiple comparison tests. Due to the increased number of mutant genotypes and the resulting large number of uninformative multiple comparisons that would arise from *post hoc* Tukey test, Holm‐corrected *t*‐tests were used to assess the FR responsiveness of Col‐0 and the *bg1*, *q1124* and *nced3/5* mutants in Fig. 4(d) (see later).

## Results

### Low‐dose UV‐B enhances stomatal opening in a UVR8‐ and phototropin‐dependent manner in seedling tissue

To assess the effect of low‐dose UV‐B (1 μmol m^−2^ s^−1^) on stomatal aperture, 7‐d‐old Arabidopsis seedlings were transferred to WL ± UV‐B at dawn (light spectra are presented in Fig. S1). Cotyledon stomatal apertures were recorded over a 6‐h period and are presented in Fig. 1(a). In contrast to reports using older leaves and/or higher doses of UV‐B or epidermal strips (He *et al*., 2013; Tossi *et al*., 2014; Li *et al*., 2017; Ge *et al*., 2020), seedlings supplemented with UV‐B showed enhanced stomatal opening when compared with WL controls during the latter part of the time course. Exposing seedlings to monochromatic UV‐B (Fig. 1b) showed that the UV‐B‐induced enhancement of stomatal opening requires a background of light within the 400–700‐nm wavelength range. Stomatal responses to UV‐B were also analysed in mature plants. Here, apertures were measured from rosette leaves, and in leaf disc and epidermal peel tissue treated while floating on a buffer. In contrast to cotyledons, no UV‐B‐enhanced stomatal opening was observed in rosette leaves (Fig. S2a–c). Some UV‐B‐mediated increase in stomatal aperture was, however, observed in leaf disc and epidermal tissue (Fig. S2d,e).

Blue light‐induced stomatal opening involves the redundant actions of phototropin photoreceptors, phot1 and phot2 (Kinoshita *et al*., 2001). Analyses of the photoreceptor mutants *uvr8‐6* and *phot1/2* showed that both are required for UV‐B‐enhanced stomatal opening (Fig. 1c,d), suggesting a role for phototropin and UVR8 signalling. Additionally, mutants deficient in the downstream phototropin signalling component BLUS1 (*blus1‐3*) (Takemiya *et al*., 2013) displayed a similar phenotype to *phot1/2* (Fig. 1e), with no additional opening in response to UV‐B supplementation observed. These data provide further support for a role for phototropin signalling in UV‐B–enhanced stomatal opening.

Following UV‐B absorption, UVR8 dimers monomerise and interact with the E3 ubiquitin ligase CONSTITUTIVELY PHOTOMORPHOGENIC (COP1) (Favory *et al*., 2009; Cloix *et al*., 2012). This stabilises the bZIP transcription factors ELONGATED HYPOCOTYL 5 (HY5) and HY5 HOMOLOGUE (HYH), which control the expression of a number of UV‐B‐regulated genes (Brown *et al*., 2005; Brown & Jenkins, 2008). We further investigated the involvement of these components in the enhanced stomatal opening response observed under UV‐B‐supplemented conditions. The *hy5/hyh* double mutant displayed wild‐type stomatal responses to UV‐B light, suggesting that these transcription factors are not required for UV‐B‐mediated stomatal opening (Fig. S3a). This mutant is in the Ws background, which behaved similarly to Col‐0, confirming the consistency of this response across different Arabidopsis accessions. Consistent with previous reports (Mao *et al*., 2005, p. 2; Khanna *et al*., 2014; An *et al*., 2022), the *cop1* mutant showed consistently open stomata under all conditions (Fig. S3b). No further opening was observed following UV‐B supplementation, but interpretation of this result is confounded by the mutant's existing phenotype. Additionally, we observed that the *tt4* mutant (a flavonoid biosynthesis mutant hypersensitive to UV‐B and more prone to UV‐B‐induced damage (Li *et al*., 1993) showed no UV‐B‐mediated enhancement of stomatal opening, suggesting damaging amounts of UV‐B may prevent the enhanced opening observed at 6 h (Fig. S3c).

### Low R : FR ratio treatment inhibits stomatal opening and is antagonised by UV‐B

**Fig. 2:**
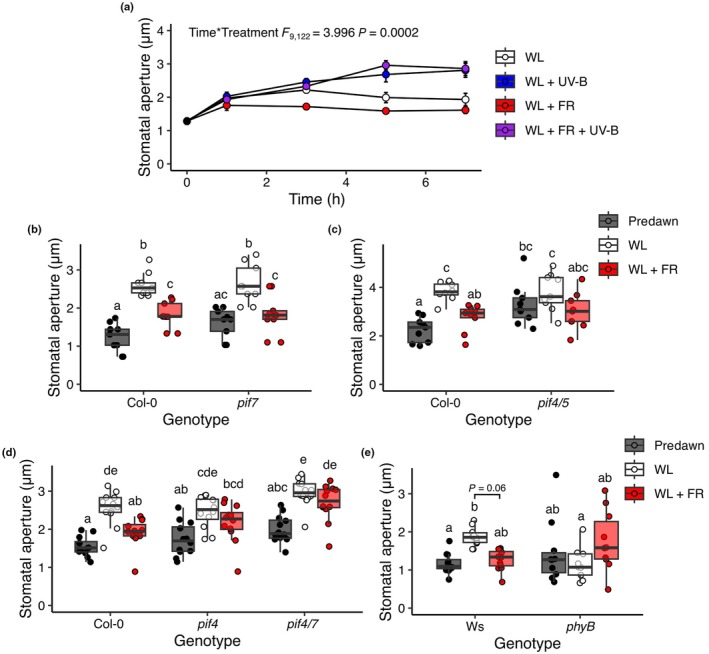
Low R : FR inhibits stomatal opening in a phytochrome interacting factor‐dependent manner and is antagonised by UV‐B. (a) Arabidopsis Col‐0 seedling stomatal apertures were monitored over a 7‐h time course where plants were treated with white light (WL) ± far red (FR) and/or low dose UV‐B. (b–e) Col‐0, Ws, *phyB* and *pif* mutant seedlings were treated with WL ± FR for 2 h before measurement of stomatal apertures. These were also measured before dawn (predawn). In (a) data are presented as the mean ± SE of each plant's average stomatal aperture. In (b, c), data are presented as boxplots showing the median and interquartile range (IQR) of each group. The upper and lower whiskers represent data within 1.5 × IQR. Each individual plant's mean stomatal aperture is represented as a point on the plot. For all genotype treatment combinations, *n* = 9–12 seedlings over three independent experiments. Each mean seedling stomatal aperture was calculated from 10 to 12 stomatal measurements. Data were analysed using a 2‐way ANOVA, followed by Tukey's multiple comparison test.

Reduced R : FR is a major component of vegetative shade that drives inactivation of phytochrome photoreceptors, auxin production and the elongation of stems for light foraging (Ballare *et al*., 1990; Franklin, 2008; Fernández‐Milmanda & Ballaré, 2021). The effect of reduced R : FR (achieved by supplementing WL with FR) on seedling stomatal apertures was investigated (Figs 2a, S2). Low R : FR decreased stomatal aperture in a response observed after 1–3 h of treatment. When WL was supplemented with both FR and UV‐B light, stomatal apertures were no longer reduced, suggesting that the presence of UV‐B overrides the effect of low R : FR. Decreased stomatal apertures were also observed in rosette leaves, although at later time points than in cotyledons (Fig. S2a–c).

To investigate whether these responses are conserved beyond Arabidopsis, the movement of the Brassica crop Chinese kale (*Brassica oleracea* var. alboglabra) cotyledon stomata was monitored in response to FR and UV‐B supplementation (Fig. S4a). The increased cotyledon size of this species additionally facilitated gas exchange analyses. In support of our Arabidopsis observations, stomatal apertures were reduced in plants treated with WL supplemented with FR for 6 h, when compared with WL controls. The addition of low‐dose UV‐B treatment also overcame the FR supplementation effect (Fig. S4a). Unlike Arabidopsis, Chinese kale did not show significant differences between WL‐ and FR‐supplemented plants at 2 h; however, both stomatal apertures and stomatal conductance trended towards decreased values (Fig. S4b,c). No significant differences in photosynthetic CO_2_ assimilation were recorded between WL‐ and FR‐supplemented plants over this time period (Fig. S4d,e).

The role of phytochrome interacting factor (PIF) transcription factors in Arabidopsis stomatal responses to low R : FR was next explored, as PIFs have been shown to regulate both shade avoidance (Lorrain *et al*., 2008; Leivar & Quail, 2011; Li *et al*., 2012) and stomatal aperture (Li *et al*., 2022; Rovira *et al*., 2024). PIF4, PIF5 and PIF7 are the major regulators of plant architectural responses to low R : FR, so we therefore focused on these genes (Lorrain *et al*., 2008; Hornitschek *et al*., 2009; Li *et al*., 2012; Burko *et al*., 2022). *pif7* mutant seedlings displayed a wild‐type response to reduced R : FR (Fig. 2b), whereas *pif4* and *pif4/5* mutants showed more variable stomatal apertures, with no significant differences between WL and low R : FR (Fig. 2c,d). Furthermore, the *pif4/7* double mutant displayed an insensitivity to low R : FR, with significantly more open stomata than Col‐0 (Fig. 2d). This suggests a dominant role for PIF4 in inhibiting stomatal opening in low R : FR conditions, with a potential minor redundant role for PIF7. Consistent with previous reports (Hayes *et al*., 2017), UV‐B supplementation reduced *PIF4* transcript accumulation in a UVR8‐dependent manner (Fig. S5a). UVR8 has also been shown to target PIF4 protein for degradation in UV‐B (Hayes *et al*., 2017; Tavridou *et al*., 2020a), presenting the possibility that PIF4 integrates low R : FR and UV‐B signalling in the regulation of stomatal aperture. The effect of UV‐B supplementation on stomatal aperture was investigated in *pif4/7* mutants at 6 h (Fig. S5b), when maximum responsiveness to UV‐B treatment was observed in wild‐type seedlings (Fig. 2a). UV‐B‐mediated increases in stomatal aperture were mediated by UVR8 at this time point (Fig. S5c). In contrast to low R : FR, responsiveness to UV‐B was retained in *pif4/7* mutants (Fig. S5b), supporting the existence of a PIF4‐independent mechanism promoting stomatal opening.

PIF activity is regulated by the red and far‐red light‐absorbing phytochrome photoreceptors (Cheng *et al*., 2021), with phyB performing a major role. In high R : FR, activated phyB binds to PIFs, leading to their ubiquitination and degradation via the 26S proteasome (Halliday & Whitelam, 2003; Monte *et al*., 2003; Franklin *et al*., 2003b). The stomatal response of *phyB* mutants to low R : FR was therefore explored (Fig. 2e). These plants displayed significantly smaller stomatal apertures than wild‐type controls in WL, consistent with a role for phyB in promoting stomatal opening (Wang *et al*., 2010). The Ws ecotype contains a natural deleterious mutation at its *PHYD* locus (Aukerman *et al*., 1997). PhyD and PhyE perform minor redundant roles with phyB in the regulation of multiple light responses (Devlin *et al*., 1999; Franklin *et al*., 2003a). It is possible that phyD also contributes to the regulation of seedling stomatal aperture, but further mutant analysis would be required to confirm this. Apertures of Ws *phyB* mutants were not further decreased in low R : FR, suggesting that phyE is not involved in this response.

To exclude the possibility that light treatments were indirectly affecting stomatal apertures through leaf temperature changes, thermal imaging was used to track cotyledon temperature over a 6‐h time course (Fig. S6a,b). At 2 h post dawn, there was no significant difference in cotyledon temperature between WL and WL + FR treatments. However, treatments involving the addition of UV‐B showed cotyledons to be *c*. 0.5°C warmer than treatments without UV‐B (Fig. S6c). Temperatures greater than 35°C have been shown to elicit increased stomatal opening (Devireddy *et al*., 2020; Kostaki *et al*., 2020; Korte *et al*., 2023). To assess whether slightly elevated cotyledon temperature may account for the increased stomatal opening observed in UV‐B supplemented conditions, seedlings were treated ± UV‐B at 20 and 28°C (Fig. S6d). Incubation at 28°C did not promote stomatal opening, and UV‐B responses were observed at both temperatures. The stomatal densities of *uvr8‐6* and *pif4* mutant cotyledons were additionally analysed and found to show no significant differences from Col‐0 (Fig. S7). It can therefore be concluded that differences in UV‐B and FR responses observed in these genotypes are not due to differences in stomatal density.

### R : FR and UV‐B control ABA levels

ABA performs a major role in regulating stomatal responses to drought stress. It is a potent trigger of stomatal closure and functions to keep stomata closed by inhibiting stomatal opening (Hsu *et al*., 2021; Pei *et al*., 2022). Multiple studies have reported links between the biosynthesis and signalling of ABA and phytochrome signalling (González *et al*., 2012; Holalu *et al*., 2020; Liang *et al*., 2020; Qi *et al*., 2020; Li *et al*., 2022). PIF and ABA accumulation dynamics have been observed to perform a role in the regulation of stomatal apertures over day : night cycles (Rovira *et al*., 2024). ABA signalling has also been linked to UV‐B responses, as in *Zea mays*, high‐dose UV‐B treatment stimulates ABA production (Tossi *et al*., 2009).

To investigate the impact of FR and/or UV‐B supplementation on seedling ABA metabolism, two pre‐existing RNA‐seq datasets from 7‐d‐old Arabidopsis seedlings treated with WL ± FR ± UV‐B for 3–4 h were re‐analysed (Tavridou *et al*., 2020b; Sharma *et al*., 2023). Genes tagged with both ABA biosynthesis (GO:0009688) and ABA catabolism (GO:0046345) show clear patterns of regulation, with UV‐B up‐ and downregulated subsets. The effect of FR supplementation alone is less clear, with a smaller number of differentially expressed genes under these conditions (Fig. S8). A similar response was observed for genes tagged with positive and negative ABA signalling GO terms (GO:0009789 and GO:0009788, respectively; Fig. S9).

**Fig. 3:**
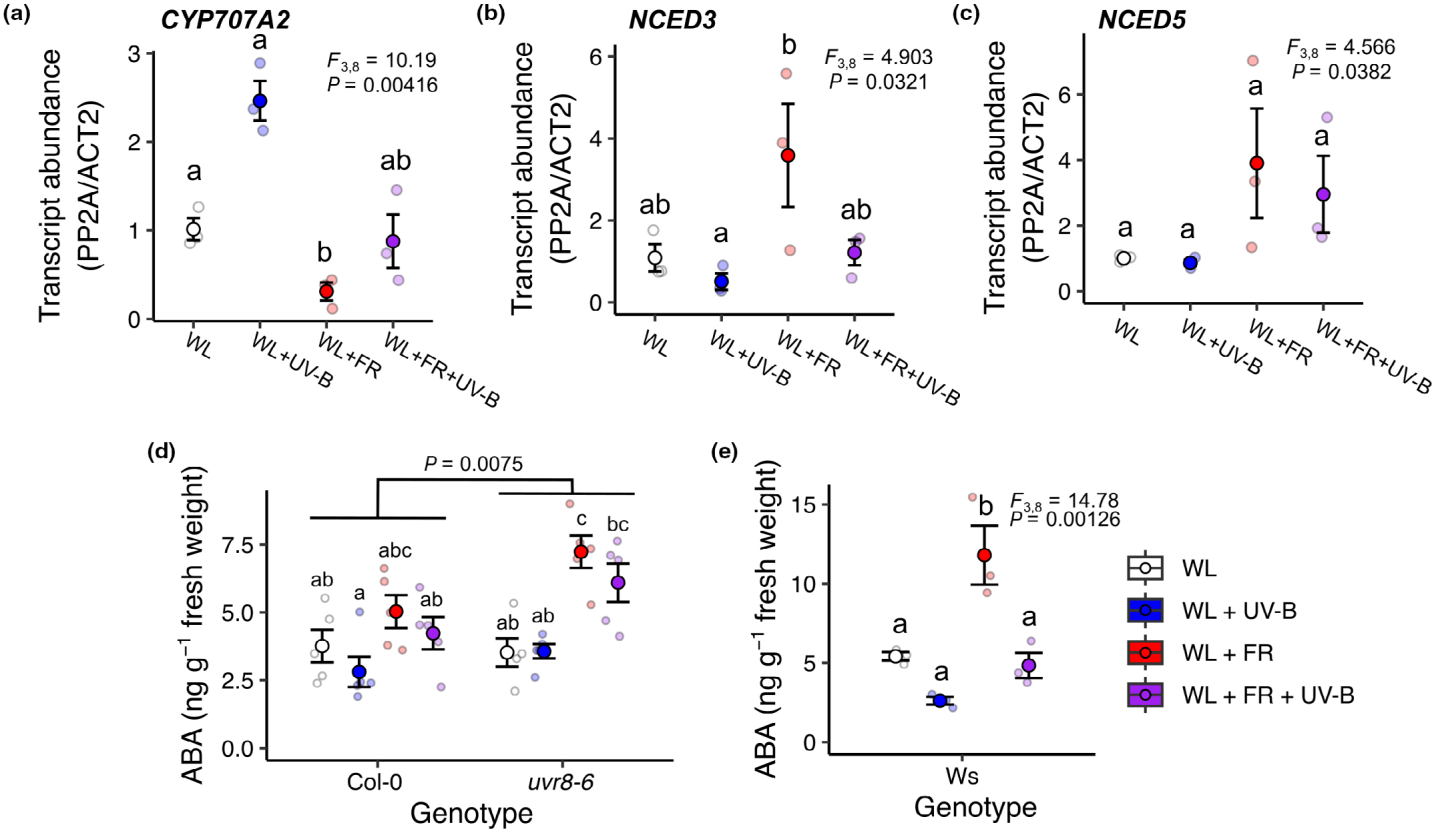
Low R : FR and UV‐B supplementation regulate the transcript abundance of key abscisic acid (ABA) metabolism enzymes and seedling ABA content. (a) *CYP707A2*, (b) *NCED3* and (c) *NCED5* transcript abundance was measured in the aerial tissue of 7‐d‐old Arabidopsis seedlings treated with white light (WL) ± far red (FR) ± UV‐B light for 6 h. ABA levels in the aerial tissue of 7‐d‐old (d) Col‐0 and *uvr8‐6*, and (e) Ws seedlings treated for 6 h with WL ± FR ± UV‐B light and quantified using LC‐MS. All data are presented as small semi‐transparent points and the mean as a larger non‐transparent point. Error bars represent SE. (a–c) *n* = 3 pooled seedling RNA samples, (d) *n* = 5 pooled seedling samples over 5 independent experiments, and (e) *n* = 3 samples over three independent experiments. All data were analysed using a 2‐way ANOVA, followed by Tukey multiple comparison tests, except for (e), where a one‐way ANOVA was used.

We next quantified the transcript abundances of key ABA biosynthesis and catabolism genes (Figs 3a–c, S10) in seedlings treated with FR and/or UV‐B supplementation for 6 h. We observed a significant effect of light treatment on the transcript accumulation of key ABA biosynthesis enzymes *NCED3* and *NCED5* (Fig. 3b,c). *Post hoc* tests showed that *NCED3* is significantly upregulated in low R : FR, whereas differences between individual groups were not observed for *NCED5*. We also analysed beta glucosidase enzyme (*BG1*) (which encodes an enzyme that rapidly generates active ABA from a pool of inactive ABA glucosyl‐ester, also known as *BGLU18*) and observed no significant effects of light treatment on transcript abundance (Fig. S10). Interestingly, a significant decrease in *BG1* transcript levels was observed following UV‐B treatment in the RNAseq analysis, potentially reflecting differences in treatment length between studies (Fig. S9). The genes *CYP707A1–3* encode ABA catabolism enzymes involved in the breakdown of ABA (Saito *et al*., 2004). Similar to the RNAseq data, significant effects of light treatment were observed on the transcript abundance of these genes. *CYP707A2* showed significant downregulation in low R : FR and higher transcript levels in UV‐B across multiple biological repeats. Conversely, *CYP707A1* and *A3* showed opposite responses, with decreased transcript abundance in UV‐B (Fig. S10).

To assess how the ABA content of aerial seedling tissue was affected by different light treatments, we assayed the ABA content of Col‐0, *uvr8‐6*, and Ws seedlings treated with UV‐B, FR or a combination of the two light treatments for 6 h. Analysis of Col‐0 and *uvr8‐6* data in Fig. 3(d) using a two‐way ANOVA showed a significant effect of both genotype (*F*_1,32_ = 8.15, *P* = 0.008) and light treatment (*F*_3,32_ = 11.45, *P* < 0.001), although no significant interaction was observed (*F*_1,3_ = 1.90, *P* = 0.150). ABA content in Col‐0 and *uvr8‐6* seedlings was quite variable, and *post hoc* tests showed no significant differences between individual groups. Much less variability was, however, observed in Ws seedlings. Here, analysis using a one‐way ANOVA showed a similar significant effect of light treatment (*F*_3,8_ = 14.78, *P* = 0.001). Furthermore, *post hoc* multiple comparison tests showed a significant increase in seedling ABA content in low R : FR, which was abolished when FR was combined with UV‐B (Fig. 3e). In both Col‐0 and Ws accessions, increased ABA content was repeatedly observed in low R : FR conditions, whereas decreased ABA was observed in the presence of UV‐B. When low R : FR and UV‐B treatments were combined, an intermediate ABA content was observed, resembling levels observed in WL. Together, these data suggest that UV‐B perceived by UVR8 can counteract low R : FR‐induced increases in seedling ABA (Fig. 3d,e).

**Fig. 4:**
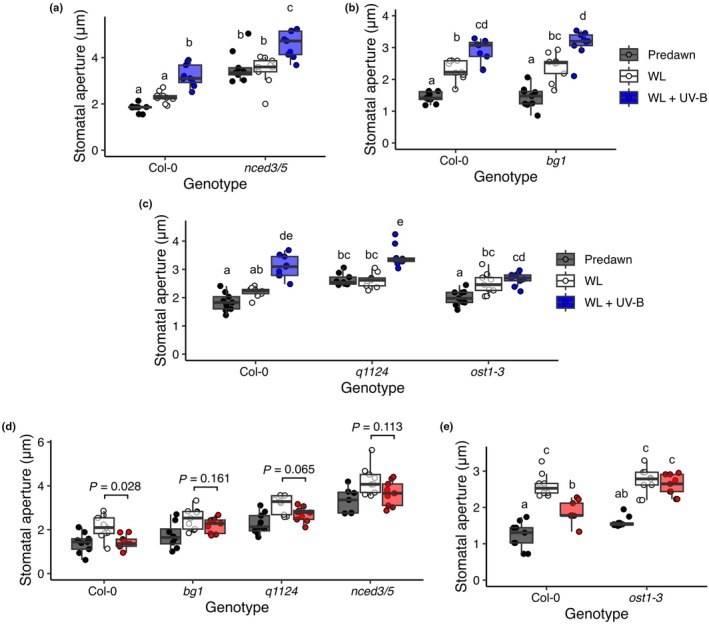
Abscisic acid (ABA) signalling and metabolism mutants respond to UV‐B but display defective responses to low R : FR. Stomatal apertures of 7‐d‐old Arabidopsis (a) ABA biosynthesis mutants *nced3/5* and (b) *bg1*, and (c) ABA signalling mutants *q1124* and *ost1‐3*, were measured predawn and following a 6 h white light (WL) ± UV‐B treatment. The stomatal aperture response of ABA biosynthesis and signalling mutants (d) *bg1*, *q1124*, *nced3/5* and (e) *ost1‐3* was measured predawn and in response to 2 h WL ± far red (FR) treatment. Data are presented as boxplots showing the median and interquartile range (IQR) of each group. The upper and lower whiskers represent data within 1.5× IQR. Each individual plant's mean stomatal aperture is represented as a point on the plot. For all genotype and treatment combinations, *n* = 9 seedlings over three independent experiments. Each mean seedling stomatal aperture was calculated from 10 stomatal measurements. All data were analysed using a 2‐way ANOVA, followed by Tukey multiple comparison tests, except for (d) where *t* tests were used to make specific comparisons and adjusted for multiple comparisons using a Holm correction. (e) *ost1‐3* was grown in parallel with *pif7‐2* (Fig. 2c) each plot uses the same Col‐0 control data.

To further investigate the potential role of ABA signalling in light‐regulated stomatal movement, mutants defective in ABA biosynthesis (*nced3/5* – defective in *de novo* ABA biosynthesis (Frey *et al*., 2012), ABA activation (*bg1*; Lee *et al*., 2006), ABA signalling (*q1124 –* a quadruple ABA receptor mutant; Park *et al*., 2009) and stomatal opening (*ost1‐3 –* a mutant in a key downstream kinase; Yoshida *et al*., 2002)) were assayed in response to UV‐B and low R : FR treatments. Here, the ABA biosynthesis mutants, *nced3/5* and *bg1*, and the quadruple receptor mutant *q1124* displayed wild‐type responses to UV‐B supplementation, whereas the *ost1‐3* mutant showed no significant increase in stomatal opening (Fig. 4a–c). The *nced3/5* ABA biosynthesis and *q1124* ABA receptor mutants displayed increased apertures in WL, supporting previous observations (Merilo *et al*., 2013; Pridgeon & Hetherington, 2021). In all ABA biosynthesis and receptor mutants, no significant differences were observed between WL and low R : FR conditions, supporting the involvement of ABA in the low R : FR‐mediated inhibition of stomatal opening (Fig. 4d). Interestingly, this phenotype was most pronounced in the *bg1* ABA biosynthesis mutant, suggesting that deconjugation of ABA from ABA‐GE is central to elevating ABA levels in low R : FR conditions (Fig. 4d). Similarly to *bg1*, the *ost1‐3* signalling mutant showed no stomatal aperture response to low R : FR conditions, confirming the importance of OST1 in stomatal aperture regulation (Yoshida *et al*., 2002) (Fig. 4e). Together, these data suggest that ABA biosynthesis and signalling are not essential for UV‐B‐induced stomatal opening but are required for low R : FR‐induced inhibition of stomatal opening.

## Discussion

This study shows that the stomatal movements of seedlings are modulated by integrated light quality signals. In both Arabidopsis and the Brassica crop Chinese Kale, low R : FR acts to limit stomatal aperture in seedling cotyledons (Fig. 2). However, when cotyledons are additionally exposed to low‐dose UV‐B, the low R : FR restriction of stomatal aperture is lifted, and stomatal opening is promoted (Figs 1, 2a). Our data suggest that low R : FR inhibits stomatal opening through promoting ABA accumulation, and that UV‐B light can antagonise this, in part, through suppression of ABA levels (Figs 3, 4).

### Low‐dose UV‐B promotes stomatal opening in seedling cotyledons

In contrast to our observations, most studies focusing on stomatal responses to UV‐B have shown UV‐B irradiation to induce stomatal closure. A number of components underlying this response have been identified, including the UVR8 photoreceptor, COP1, HY5 and HYH (Tossi *et al*., 2014; Ge *et al*., 2020); a G alpha protein (He *et al*., 2013); MAP KINASES (MAPKs) (Li *et al*., 2017); ethylene (He *et al*., 2011; Ge *et al*., 2020); hydrogen peroxide (H_2_O_2_); nitric oxide (NO) (He *et al*., 2005, 2013); and the LIPOXYGENASE 1 (LOX1) enzyme (Liu *et al*., 2025). The latter are stress signalling components, most likely induced by the high doses of UV‐B applied. Here, we found that lower dose UV‐B (1 μmol m^−2^ s^−1^) applied in a background of WL increases stomatal apertures of the cotyledon abaxial epidermis (Figs 1, 2a). This occurs in both Col‐0 and Ws Arabidopsis accessions and Chinese Kale (Figs 1, S3, S4a), but not in mature plant leaves (Fig. S2a–c). However, caution must be applied when comparing responses of cotyledons and mature rosette leaves. Many physiological factors differ between the two, including the UV‐B penetrance of cotyledon and mature plant leaf tissue. When stomata are isolated from the rest of the leaf (epidermal strips) or leaf tissue is excised from the plant (leaf discs), a small amount of opening is observed (Fig. S2d,e), suggesting that although isolated guard cells can respond to low‐dose UV‐B supplementation, other cells are required for a full opening response. It is unlikely that the light treatments used in this study were damaging, as *F*_v_/*F*_m_ measurements of Chl fluorescence (commonly used as an indicator of photosystem health (Murchie & Lawson, 2013)) varied little in response to low R : FR or UV‐B. Unsurprisingly, a mild reduction in *F*_v_/*F*_m_ was observed in UV‐B‐treated *uvr8‐6* mutants, which are unable to initiate photoprotective responses (Fig. S11). Some studies have reported stomatal opening in response to UV‐B supplementation in *Vicia faba* (Jansen & Van Den Noort, 2000), cucumber (Teramura, 1983) and certain Ericaceae species (Musil & Wand, 1993), and it has been suggested that the effect of UV‐B light on stomata is dependent on the plant metabolic state (Nogués *et al*., 1999; Jansen & Van Den Noort, 2000).

We show that the opening of seedling abaxial stomata following UV‐B treatment requires the presence of light within the photosynthetically active range (400–700 nm), functional phototropin and UVR8 photoreceptors (Fig. 1), together with the downstream phototropin signalling component BLUS1 (Fig. 1e). The requirement for phototropin signalling differs from observations showing that low doses of UV‐B alone could stimulate stomatal opening in both Arabidopsis and *Vicia faba* (Eisinger *et al*., 2003). However, there are numerous differences between plant growth conditions, ages and experimental procedures that may explain the contrasting observations. In this study, we have focused on the stomatal apertures of intact seedling cotyledon tissue and tracked apertures over a longer period through the course of the day.

Our data suggest that UV‐B‐mediated promotion of stomatal opening does not require the UV‐B signalling components HY5 or HYH (Fig. S3a). This is in contrast to the UVR8‐mediated promotion of stomatal closure (Tossi *et al*., 2014; Ge *et al*., 2020). Upon assaying mutants deficient in the UV‐B signalling component, COP1, we observed a constitutively open response before dawn, and no difference between WL and WL + UV‐B conditions (Fig. S3b). These mutants have previously been shown to present a constitutively open stomata phenotype in the dark (Mao *et al*., 2005; Wang *et al*., 2010; Khanna *et al*., 2014; An *et al*., 2022), confounding interpretation of the role of COP1 in UV‐B promotion of stomatal opening.

### Low R : FR inhibits cotyledon stomatal opening in a PIF4‐dependent manner

We further show that seedlings exposed to low R : FR ratio display reduced stomatal apertures following 1–3 h of treatment in a response requiring phyB and PIF4 (Figs 2, 3, S2). Previous studies have shown low R : FR to inhibit stomatal opening in *Commelina communis* and orchids (Roth‐Bejerano *et al*., 1990; Talbott *et al*., 2002), with no effect observed in Arabidopsis (Talbott *et al*., 2003). The differences in our observations may be due to using intact cotyledon tissue, as opposed to epidermal peels from mature leaves. Monochromatic FR treatment has been shown to reduce stomatal apertures in rice in a mechanism requiring the PIF‐homologue, OsPIL15 (Li *et al*., 2022). PIFs have also been shown to play a role in regulating the daily rhythmic opening and closing of stomata (Rovira *et al*., 2024). Here, the authors show PIFs to act in opposition to ABA, promoting the accumulation of K^+^ import channel POTASSIUM CHANNEL IN ARABIDOPSIS THALIANA (KAT1) and ultimately stomatal opening at dawn in 3‐d‐old Arabidopsis seedlings. This positive role in stomatal opening contrasts with the negative role identified in this study and may reflect differences in plant age or growth conditions. Here, seedlings were soil‐grown, whereas Rovira *et al*. used ½ Murashige & Skoog (½ MS) plates, where humidity would be considerably higher. Growth under high humidity conditions is known to affect stomatal responses to several signals, including ABA (Aliniaeifard & Van Meeteren, 2013). KAT1 activity depends on its cellular location. ABA can trigger endocytosis of KAT1 from guard cell plasma membranes to endosomal compartments, thus preventing it from contributing to stomatal opening (Sutter *et al*., 2007). We observed a (non‐significant) increase in *KAT1* transcript abundance in low R : FR (Fig. S10), which may involve PIFs (Rovira *et al*., 2024). The functional relevance of increased *KAT1* abundance in low R : FR is, however, unclear, as the increased ABA levels in these conditions would likely reduce KAT1 activity. Further work is therefore required to explore the potential role of KAT1 in this response.

Several studies have identified connections between ABA signalling, phytochrome signalling and red/far‐red light responses. PIFs have been shown to bind to the promoter regions of ABA biosynthesis and signalling genes (Liang *et al*., 2020), as well as interact with the ABA receptor proteins PYL8 and PYL9 (Qi *et al*., 2020). Mutants deficient in phyB show increased ABA levels but reduced ABA sensitivity in well‐watered conditions (González *et al*., 2012). ABA content has been shown to decrease in plants treated with red light (Zhu *et al*., 2020), and conversely, increase in low R : FR (Cagnola *et al*., 2012; González‐Grandío *et al*., 2013; Holalu & Finlayson, 2017; Ortiz‐Alcaide *et al*., 2019; Holalu *et al*., 2020; Michaud *et al*., 2023). Additionally, genes tagged with the GO term ‘response to ABA’ are upregulated in low R : FR‐treated Arabidopsis seedlings (Kohnen *et al*., 2016) and FR‐treated leaf tips (Küpers *et al*., 2023).

Analysis of RNAseq data from experiments using similar treatments to this study (Tavridou *et al*., 2020b; Sharma *et al*., 2023) showed that UV‐B (and to a smaller extent FR) supplementation altered the transcript abundance of genes tagged with ABA metabolism and signalling GO terms (Figs S8, S9). In our conditions, transcripts of the ABA biosynthesis enzyme NCED3 were upregulated, while transcripts of the ABA degradation enzyme CYP707A2 were downregulated by FR, but not by FR + UV‐B supplementation (Fig. 3). We observed multiple ABA signalling and metabolism mutants to show reduced responses to low R : FR (Fig. 4). These data support a mechanism whereby, in low R : FR, expression of the ABA biosynthesis enzyme NCED3 is increased and BG1 functions to generate active ABA from a pool of inactive ABA‐GE, which together increase ABA signalling, OST1 activation and inhibition of stomatal opening. The parallel decrease in ABA degradation enzyme *CYP707A2* transcripts in these conditions may further contribute to increased seedling ABA content.

### UV‐B antagonises ABA accumulation and stomatal responses to low R : FR

**Fig. 5:**
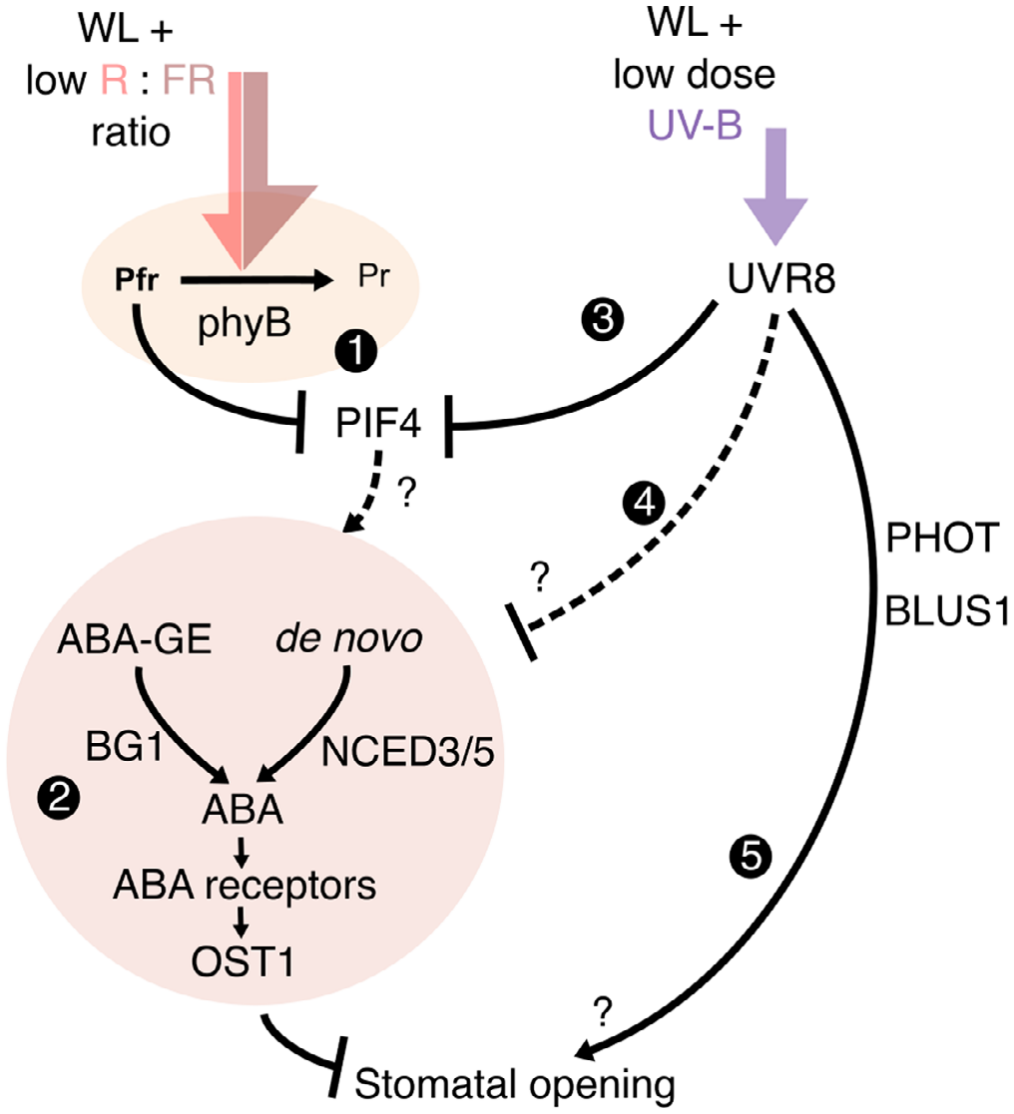
Proposed model for seedling stomatal aperture control by low R : FR and UV‐B. A proposed mechanism for the action of low‐red : far‐red (R : FR) and UV‐B on stomatal opening. (1) Low R : FR promotes the conversion of the phytochrome B photoreceptor to its inactive Pr state, which, in turn, stabilises the phytochrome interacting factors (PIFs). In this study, we observe PHYTOCHROME INTERACTING FACTOR 4 (PIF4) is required for seedling stomatal responses to FR supplementation. (2) Abscisic acid (ABA) biosynthesis and signalling components are required for inhibition of stomatal opening, likely downstream of PIF activity. The beta glucosidase enzyme (BG1) is involved in cleaving the glucose ester off an inactive pool of ABA‐GE to rapidly generate active ABA, and the NCED3 and NCED5 enzymes catalyse a key step in *de novo* ABA biosynthesis. Here, we suggest that low R : FR‐induced increases in seedling ABA levels drive inhibition of stomatal opening. UV‐B supplementation abolishes the inhibition of stomatal opening in low R : FR. (3) UV‐B, perceived by UV RESISTANCE LOCUS 8 (UVR8) targets PIFs for inactivation and/or degradation. Inhibition of PIF function likely prevents the accumulation of ABA, relieving inhibition of stomatal opening. (4) UVR8 may additionally function to directly inhibit ABA biosynthesis and/or signalling. (5) UVR8 also promotes further stomatal opening through additional mechanisms unrelated to ABA signalling, involving phototropin photoreceptors and BLUE LIGHT SIGNALLING 1 (BLUS1). Solid and dashed lines represent known and unknown connections respectively.

When seedlings were exposed to both FR and UV‐B supplementation simultaneously, UV‐B antagonised low R : FR‐mediated inhibition of stomatal opening (Fig. 2a). A parallel response is observed in hypocotyl elongation, where low R : FR‐induced elongation is inhibited in the presence of UV‐B. This results, in part, from UVR8‐mediated sequestration of COP1, destabilising PIF proteins, independently from HY5/HYH‐mediated signalling pathways (Hayes *et al*., 2014; Sharma *et al*., 2019). Addition of UV‐B to low R : FR reversed elevations in ABA (Fig. 3d,e), likely through the upregulation of *CYP707A2* and downregulation of *NCED3* (Fig. 3a,b). These observations, coupled with analyses of ABA biosynthesis and signalling mutants (Fig. 4d,e), suggest that an increase in ABA content is required for low R : FR‐mediated inhibition of stomatal opening. A hypothetical model outlining the mechanism behind this response is proposed in Fig. 5. In vegetational shade, low R : FR inactivates phyB, stabilising and promoting PIF4 activity. ABA accumulates via *de novo* synthesis and de‐conjugation of ABA‐GE. When plants reach a gap in the canopy, UV‐B perceived by UVR8 suppresses PIF4 activity (Hayes *et al*., 2014; Sharma *et al*., 2019) and ABA accumulation, removing repression of stomatal opening. In non‐shaded (high R : FR) conditions, UV‐B supplementation decreases ABA content in a UVR8‐dependent manner and promotes stomatal opening through a mechanism requiring phototropins (Fig. 3e).

### Conclusion: seedlings integrate light quality signals to coordinate growth and stomatal aperture in dynamic light environments, via regulation of hormone abundance

Following germination, seedlings must balance growth and water use to transition to photoautotrophic development without depleting available resources. Light quality provides key information concerning the prevailing levels of vegetational shade and sunlight availability (Fernández‐Milmanda & Ballaré, 2021). Plants perceive light quality using multiple photoreceptors, and integrate this information to regulate levels of multiple hormones, altering their physiology and development to optimise survival (Brini *et al*., 2022). Low R : FR and UV‐B control auxin and gibberellin abundance and signalling to adjust plant architecture for maximum light capture. Here, we show that low R : FR and UV‐B also regulate ABA content in seedling aerial tissue to control stomatal movements.

While the data presented here were gathered from plants grown in controlled environment conditions and have direct relevance to protected crops, it is pertinent to ask whether they have wider relevance to the field situation. In this context, it is worth considering that when a seedling emerges under a vegetative canopy, low R : FR‐mediated inhibition of stomatal opening may allow seedlings to restrict water loss without greatly impacting photosynthetic rates. While testing this possibility is beyond the scope of the current work, it seems likely that the low R : FR‐mediated inhibition of opening would be overridden in the presence of low amounts of UV‐B light. This would allow seedling stomata to rapidly respond to sunflecks (gaps in the canopy known to contain quantities of UV‐B light) (Moriconi *et al*., 2018). When seedlings emerge from a canopy, they are exposed to increased amounts of photosynthetic light. R : FR increases, and the inhibition of stomatal opening is likely attenuated. Prolonged exposure to ambient UV‐B would then further open stomata, ensuring maximum photosynthetic productivity in sunlight. Additional experiments carried out in the field are required to test this possibility directly. However, it should be noted that the relevance of our experiments to other species, at least in the Brassicaceae, is strengthened by our findings in two members of this family.

## Competing interests

None declared.

## Author contributions

MG, KAF and AJP designed experiments. MG, LH and AJP performed experiments and analysed data. MG, KAF and AJP wrote the manuscript.

## Disclaimer

The New Phytologist Foundation remains neutral with regard to jurisdictional claims in maps and in any institutional affiliations.

## Supporting information


**Fig. S1** Light spectra of the different treatments described in this study.
**Fig. S2** *Cot*yledons and rosette leaves differ in stomatal responses to light quality.
**Fig. S3** *UV‐B‐*mediated stomatal opening likely requires COP1, but not HY5/HYH.
**Fig. S4** *Low d*ose UV‐B treatment overrides far‐red inhibition of opening in Chinese kale cotyledons.
**Fig. S5** UV‐B regulates PIF4 transcript abundance and induces stomatal opening in pif4/7 mutants but not uvr8‐6.
**Fig. S6** UV‐B‐mediated increases in stomatal aperture do not result from small elevations in cotyledon temperature.
**Fig. S7** uvr8 and pif4 mutants show no significant differences in cotyledon stomatal density.
**Fig. S8** *F*R and UV‐B supplementation affect the transcript levels of genes involved in ABA metabolism.
**Fig. S9** FR and UV‐B supplementation affect the transcript levels of genes involved in ABA signalling.
**Fig. S10** QPCR of candidate low R : FR and low dose UV‐B response genes.
**Fig. S11** Low dose UV‐B supplementation has only minor effects on maximum photosystem II efficiency.

**Table S1** A list of Arabidopsis lines used in this study.

**Table S2** A list of qPCR primers used in this study.
Please note: Wiley is not responsible for the content or functionality of any Supporting Information supplied by the authors. Any queries (other than missing material) should be directed to the *New Phytologist* Central Office.

## Data Availability

The data that support the findings of this study are available in the article and in Figs S1–S11 and Tables S1, S2. The RNAseq data from Tavridou *et al*. (2020b) and Sharma *et al*. (2023) were downloaded from the NCBI GEO database under accession numbers GSE146125 and GSE192469, respectively.

## References

[nph70746-bib-0001] Aliniaeifard S , Van Meeteren U . 2013. Can prolonged exposure to low VPD disturb the ABA signalling in stomatal guard cells? Journal of Experimental Botany 64: 3551–3566. 23956410 10.1093/jxb/ert192 PMC3745724

[nph70746-bib-0002] An Y‐Y , Li J , Feng Y‐X , Sun Z‐M , Li Z‐Q , Wang X‐T , Zhang M‐X , He J‐M . 2022. COP1 mediates dark‐induced stomatal closure by suppressing FT, TSF and SOC1 expression to promote NO accumulation in Arabidopsis guard cells. International Journal of Molecular Sciences 23: 15037. 36499365 10.3390/ijms232315037 PMC9736015

[nph70746-bib-0003] Aukerman MJ , Hirschfeld M , Wester L , Weaver M , Clack T , Amasino RM , Sharrock RA . 1997. A deletion in the PHYD gene of the Arabidopsis Wassilewskija ecotype defines a role for phytochrome D in red/far‐red light sensing. Plant Cell 9: 1317–1326. 9286109 10.1105/tpc.9.8.1317 PMC157000

[nph70746-bib-0004] Ballare CL , Scopel AL , Sanchez RA . 1990. Far‐red radiation reflected from adjacent leaves: an early signal of competition in plant canopies. Science 247: 329–332. 17735851 10.1126/science.247.4940.329

[nph70746-bib-0005] Brini F , Mseddi K , Brestic M , Landi M . 2022. Hormone‐mediated plant responses to light quality and quantity. Environmental and Experimental Botany 202: 105026.

[nph70746-bib-0006] Brown BA , Cloix C , Jiang GH , Kaiserli E , Herzyk P , Kliebenstein DJ , Jenkins GI . 2005. A UV‐B‐specific signaling component orchestrates plant UV protection. Proceedings of the National Academy of Sciences, USA 102: 18225–18230. 10.1073/pnas.0507187102 PMC1312397 16330762

[nph70746-bib-0007] Brown BA , Jenkins GI . 2008. UV‐B signaling pathways with different fluence‐rate response profiles are distinguished in mature Arabidopsis leaf tissue by requirement for UVR8, HY5, and HYH. Plant Physiology 146: 323–324. 10.1104/pp.107.108456 PMC2245850 18055587

[nph70746-bib-0008] Burko Y , Willige BC , Seluzicki A , Novák O , Ljung K , Chory J . 2022. PIF7 is a master regulator of thermomorphogenesis in shade. Nature Communications 13: 4942. 10.1038/s41467-022-32585-6 PMC9424238 36038577

[nph70746-bib-0009] Cagnola JI , Ploschuk E , Benech‐Arnold T , Finlayson SA , Casal JJ . 2012. Stem transcriptome reveals mechanisms to reduce the energetic cost of shade‐avoidance responses in tomato. Plant Physiology 160: 1110–1119. 22872775 10.1104/pp.112.201921 PMC3461533

[nph70746-bib-0010] Cheng M‐C , Kathare PK , Paik I , Huq E . 2021. Phytochrome signaling networks. Annual Review of Plant Biology 72: 217–244. 10.1146/annurev-arplant-080620-024221 PMC10988782 33756095

[nph70746-bib-0011] Cloix C , Kaiserli E , Heilmann M , Baxter KJ , Brown BA , O'Hara A , Smith BO , Christie JM , Jenkins GI . 2012. C‐terminal region of the UV‐B photoreceptor UVR8 initiates signaling through interaction with the COP1 protein. Proceedings of the National Academy of Sciences, USA 109: 16366–16370. 10.1073/pnas.1210898109 PMC3479605 22988111

[nph70746-bib-0012] Devireddy AR , Arbogast J , Mittler R . 2020. Coordinated and rapid whole‐plant systemic stomatal responses. New Phytologist 225: 21–25. 31454419 10.1111/nph.16143

[nph70746-bib-0099] Devlin PF , Robson PRH , Patel SR , Goosey L , Sharrock RA , Whitelam GC . 1999. Phytochrome D acts in the shade‐avoidance syndrome in Arabidopsis by controlling elongation growth and flowering time. Plant Physiology 119: 909–916. 10069829 10.1104/pp.119.3.909 PMC32105

[nph70746-bib-0013] Eisinger WR , Bogomolni RA , Taiz L . 2003. Interactions between a blue‐green reversible photoreceptor and a separate UV‐B receptor in stomatal guard cells. American Journal of Botany 90: 1560–1566. 21653331 10.3732/ajb.90.11.1560

[nph70746-bib-0014] Favory J‐J , Stec A , Gruber H , Rizzini L , Oravecz A , Funk M , Albert A , Cloix C , Jenkins GI , Oakeley EJ *et al*. 2009. Interaction of COP1 and UVR8 regulates UV‐B‐induced photomorphogenesis and stress acclimation in Arabidopsis. EMBO Journal 28: 591–601. 19165148 10.1038/emboj.2009.4 PMC2657586

[nph70746-bib-0015] Fenner M , Thompson K . 2005. The ecology of seeds. Cambridge, UK: Cambridge University Press.

[nph70746-bib-0016] Fernández‐Milmanda GL , Ballaré CL . 2021. Shade avoidance: expanding the color and hormone palette. Trends in Plant Science 26: 509–523. 33461868 10.1016/j.tplants.2020.12.006

[nph70746-bib-0017] Flütsch S , Santelia D . 2021. Mesophyll‐derived sugars are positive regulators of light‐driven stomatal opening. New Phytologist 230: 1754–1760. 33666260 10.1111/nph.17322

[nph70746-bib-0018] Franklin KA . 2008. Shade avoidance. New Phytologist 179: 930–944. 18537892 10.1111/j.1469-8137.2008.02507.x

[nph70746-bib-0019] Franklin KA , Davis SJ , Stoddart WM , Vierstra RD , Whitelam GC . 2003a. Mutant analyses define multiple roles for phytochrome c in arabidopsis photomorphogenesis. Plant Cell 15: 1981–1989. 12953105 10.1105/tpc.015164 PMC181325

[nph70746-bib-0020] Franklin KA , Praekelt U , Stoddart WM , Billingham OE , Halliday KJ , Whitelam GC . 2003b. Phytochromes B, D, and E act redundantly to control multiple physiological responses in Arabidopsis. Plant Physiology 131: 1340–1346. 12644683 10.1104/pp.102.015487 PMC166893

[nph70746-bib-0021] Frey A , Effroy D , Lefebvre V , Seo M , Perreau F , Berger A , Sechet J , To A , North HM , Marion‐Poll A . 2012. Epoxycarotenoid cleavage by NCED5 fine‐tunes ABA accumulation and affects seed dormancy and drought tolerance with other NCED family members: functional analysis of the NCED5 gene. The Plant Journal 70: 501–512. 22171989 10.1111/j.1365-313X.2011.04887.x

[nph70746-bib-0022] Ge X‐M , Hu X , Zhang J , Huang Q‐M , Gao Y , Li Z‐Q , Li S , He J‐M . 2020. UV RESISTANCE LOCUS8 mediates ultraviolet‐B‐induced stomatal closure in an ethylene‐dependent manner. Plant Science 301: 110679. 33218642 10.1016/j.plantsci.2020.110679

[nph70746-bib-0023] González CV , Ibarra SE , Piccoli PN , Botto JF , Boccalandro HE . 2012. Phytochrome B increases drought tolerance by enhancing ABA sensitivity in *Arabidopsis thaliana* . Plant, Cell & Environment 35: 1958–1968. 10.1111/j.1365-3040.2012.02529.x 22553988

[nph70746-bib-0024] González‐Grandío E , Poza‐Carrión C , Sorzano COS , Cubas P . 2013. *BRANCHED1* promotes axillary bud dormancy in response to shade in *Arabidopsis* . Plant Cell 25: 834–850. 23524661 10.1105/tpc.112.108480 PMC3634692

[nph70746-bib-0025] Halliday KJ , Whitelam GC . 2003. Changes in photoperiod or temperature alter the functional relationships between phytochromes and reveal roles for phyD and phyE. Plant Physiology 131: 1913–1920. 12692350 10.1104/pp.102.018135 PMC166947

[nph70746-bib-0026] Hayes S , Sharma A , Fraser DP , Trevisan M , Cragg‐Barber CK , Tavridou E , Fankhauser C , Jenkins GI , Franklin KA . 2017. UV‐B perceived by the UVR8 photoreceptor inhibits plant thermomorphogenesis. Current Biology 27: 120–127. 27989670 10.1016/j.cub.2016.11.004 PMC5226890

[nph70746-bib-0027] Hayes S , Velanis CN , Jenkins GI , Franklin KA . 2014. UV‐B detected by the UVR8 photoreceptor antagonizes auxin signaling and plant shade avoidance. Proceedings of the National Academy of Sciences, USA 111: 11894–11899. 10.1073/pnas.1403052111 PMC4136589 25071218

[nph70746-bib-0028] He J , Yue X , Wang R , Zhang Y . 2011. Ethylene mediates UV‐B‐induced stomatal closure via peroxidase‐dependent hydrogen peroxide synthesis in *Vicia faba* L. Journal of Experimental Botany 62: 2657–2666. 21212297 10.1093/jxb/erq431

[nph70746-bib-0029] He J‐M , Ma X‐G , Zhang Y , Sun T‐F , Xu F‐F , Chen Y‐P , Liu X , Yue M . 2013. Role and interrelationship of Gα protein, hydrogen peroxide, and nitric oxide in ultraviolet B‐induced stomatal closure in Arabidopsis leaves. Plant Physiology 161: 1570–1583. 23341360 10.1104/pp.112.211623 PMC3585617

[nph70746-bib-0030] He J‐M , Xu H , She X‐P , Song X‐G , Zhao W‐M . 2005. The role and the interrelationship of hydrogen peroxide and nitric oxide in the UV‐B‐induced stomatal closure in broad bean. Functional Plant Biology 32: 237–247. 32689127 10.1071/FP04185

[nph70746-bib-0031] Holalu SV , Finlayson SA . 2017. The ratio of red light to far red light alters *Arabidopsis* axillary bud growth and abscisic acid signalling before stem auxin changes. Journal of Experimental Botany 68: erw479. 10.1093/jxb/erw479 PMC5444464 28062593

[nph70746-bib-0032] Holalu SV , Reddy SK , Blackman BK , Finlayson SA . 2020. Phytochrome interacting factors 4 and 5 regulate axillary branching via bud abscisic acid and stem auxin signalling. Plant, Cell & Environment 43: 2224–2238. 10.1111/pce.13824 32542798

[nph70746-bib-0033] Holm M , Ma L‐G , Qu L‐J , Deng X‐W . 2002. Two interacting bZIP proteins are direct targets of COP1‐mediated control of light‐dependent gene expression in *Arabidopsis* . Genes & Development 16: 1247–1259. 12023303 10.1101/gad.969702 PMC186273

[nph70746-bib-0034] Hornitschek P , Lorrain S , Zoete V , Michielin O , Fankhauser C . 2009. Inhibition of the shade avoidance response by formation of non‐DNA binding bHLH heterodimers. EMBO Journal 28: 3893–3902. 19851283 10.1038/emboj.2009.306 PMC2797054

[nph70746-bib-0035] Hsu P , Dubeaux G , Takahashi Y , Schroeder JI . 2021. Signaling mechanisms in abscisic acid‐mediated stomatal closure. The Plant Journal 105: 307–321. 33145840 10.1111/tpj.15067 PMC7902384

[nph70746-bib-0036] Inoue S‐I , Kinoshita T . 2017. Blue light regulation of stomatal opening and the plasma membrane H+‐ATPase. Plant Physiology 174: 531–538. 28465463 10.1104/pp.17.00166 PMC5462062

[nph70746-bib-0037] Jansen MAK , Van Den Noort RE . 2000. Ultraviolet‐B radiation induces complex alterations in stomatal behaviour. Physiologia Plantarum 110: 189–194.

[nph70746-bib-0038] Kagawa T , Sakai T , Suetsugu N , Oikawa K , Ishiguro S , Kato T , Tabata S , Okada K , Wada M . 2001. *Arabidopsis* NPL1: A phototropin homolog controlling the chloroplast high‐light avoidance response. Science 291: 2138–2141. 11251116 10.1126/science.291.5511.2138

[nph70746-bib-0039] Khanna R , Li J , Tseng T‐S , Schroeder JI , Ehrhardt DW , Briggs WR . 2014. COP1 jointly modulates cytoskeletal processes and electrophysiological responses required for stomatal closure. Molecular Plant 7: 1441–1454. 25151660 10.1093/mp/ssu065 PMC4153439

[nph70746-bib-0040] Kinoshita T , Doi M , Suetsugu N , Kagawa T , Wada M , Shimazaki K . 2001. phot1 and phot2 mediate blue light regulation of stomatal opening. Nature 414: 656–660. 11740564 10.1038/414656a

[nph70746-bib-0041] Kohnen MV , Schmid‐Siegert E , Trevisan M , Petrolati LA , Sénéchal F , Müller‐Moulé P , Maloof J , Xenarios I , Fankhauser C . 2016. Neighbor detection induces organ‐specific transcriptomes, revealing patterns underlying hypocotyl‐specific growth. Plant Cell 28: 2889–2904. 27923878 10.1105/tpc.16.00463 PMC5240736

[nph70746-bib-0042] Korte P , Unzner A , Damm T , Berger S , Krischke M , Mueller MJ . 2023. High triacylglycerol turnover is required for efficient opening of stomata during heat stress in Arabidopsis. The Plant Journal 115: 81–96. 36976526 10.1111/tpj.16210

[nph70746-bib-0043] Kostaki K‐I , Coupel‐Ledru A , Bonnell VC , Gustavsson M , Sun P , McLaughlin FJ , Fraser DP , McLachlan DH , Hetherington AM , Dodd AN *et al*. 2020. Guard cells integrate light and temperature signals to control stomatal aperture. Plant Physiology 182: 1404–1419. 31949030 10.1104/pp.19.01528 PMC7054865

[nph70746-bib-0044] Küpers JJ , Snoek BL , Oskam L , Pantazopoulou CK , Matton SEA , Reinen E , Liao C‐Y , Eggermont EDC , Weekamp H , Biddanda‐Devaiah M *et al*. 2023. Local light signaling at the leaf tip drives remote differential petiole growth through auxin‐gibberellin dynamics. Current Biology 33: 75–85.e5. 36538931 10.1016/j.cub.2022.11.045 PMC9839380

[nph70746-bib-0045] Lawson T , Matthews J . 2020. Guard cell metabolism and stomatal function. Annual Review of Plant Biology 71: 273–302. 10.1146/annurev-arplant-050718-100251 32155341

[nph70746-bib-0046] Leck MA , Parker VT , Simpson R . 2008. Seedling ecology and evolution. Cambridge, UK: Cambridge University Press.

[nph70746-bib-0047] Lee KH , Piao HL , Kim H‐Y , Choi SM , Jiang F , Hartung W , Hwang I , Kwak JM , Lee I‐J , Hwang I . 2006. Activation of glucosidase via stress‐induced polymerization rapidly increases active pools of abscisic acid. Cell 126: 1109–1120. 16990135 10.1016/j.cell.2006.07.034

[nph70746-bib-0048] Leivar P , Monte E , Al‐Sady B , Carle C , Storer A , Alonso JM , Ecker JR , Quail PH . 2008a. The Arabidopsis phytochrome‐interacting factor PIF7, together with PIF3 and PIF4, regulates responses to prolonged red light by modulating phyB levels. Plant Cell 20: 337–352. 18252845 10.1105/tpc.107.052142 PMC2276449

[nph70746-bib-0049] Leivar P , Monte E , Oka Y , Liu T , Carle C , Castillon A , Huq E , Quail PH . 2008b. Multiple phytochrome‐interacting bHLH transcription factors repress premature seedling photomorphogenesis in darkness. Current Biology 18: 1815–1823. 19062289 10.1016/j.cub.2008.10.058 PMC2651225

[nph70746-bib-0050] Leivar P , Quail PH . 2011. PIFs: pivotal components in a cellular signaling hub. Trends in Plant Science 16: 19–28. 20833098 10.1016/j.tplants.2010.08.003 PMC3019249

[nph70746-bib-0051] Li F‐C , Wang J , Wu M‐M , Fan C‐M , Li X , He J‐M . 2017. Mitogen‐activated protein kinase phosphatases affect UV‐B‐induced stomatal closure via controlling NO in guard cells. Plant Physiology 173: 760–770. 27837091 10.1104/pp.16.01656 PMC5210765

[nph70746-bib-0052] Li J , Ou‐Lee TM , Raba R , Amundson RG , Last RL . 1993. arabidopsis flavonoid mutants are hypersensitive to UV‐B irradiation. Plant Cell 5: 171–179. 12271060 10.1105/tpc.5.2.171 PMC160260

[nph70746-bib-0053] Li L , Ljung K , Breton G , Schmitz RJ , Pruneda‐Paz J , Cowing‐Zitron C , Cole BJ , Ivans LJ , Pedmale UV , Jung H‐S *et al*. 2012. Linking photoreceptor excitation to changes in plant architecture. Genes & Development 26: 785–790. 22508725 10.1101/gad.187849.112 PMC3337452

[nph70746-bib-0054] Li Q , Zhou L , Chen Y , Xiao N , Zhang D , Zhang M , Wang W , Zhang C , Zhang A , Li H *et al*. 2022. Phytochrome interacting factor regulates stomatal aperture by coordinating red light and abscisic acid. Plant Cell 34: 4293–4312. 35929789 10.1093/plcell/koac244 PMC9614506

[nph70746-bib-0055] Liang S , Gao X , Wang Y , Zhang H , Yin K , Chen S , Zhang M , Zhao R . 2020. Phytochrome‐interacting factors regulate seedling growth through ABA signaling. Biochemical and Biophysical Research Communications 526: 1100–1105. 32307082 10.1016/j.bbrc.2020.04.011

[nph70746-bib-0056] Liu Y , Wang J , Liu X , Liao T , Ren H , Liu L , Huang X . 2025. The UV‐B photoreceptor UVR8 interacts with the LOX1 enzyme to promote stomatal closure through the LOX‐derived oxylipin pathway. Plant Cell 37: koaf060. 40123505 10.1093/plcell/koaf060 PMC11979336

[nph70746-bib-0057] Lorrain S , Allen T , Duek PD , Whitelam GC , Fankhauser C . 2008. Phytochrome‐mediated inhibition of shade avoidance involves degradation of growth‐promoting bHLH transcription factors. The Plant Journal 53: 312–323. 18047474 10.1111/j.1365-313X.2007.03341.x

[nph70746-bib-0058] de Lucas M , Davière J‐M , Rodríguez‐Falcón M , Pontin M , Iglesias‐Pedraz JM , Lorrain S , Fankhauser C , Blázquez MA , Titarenko E , Prat S . 2008. A molecular framework for light and gibberellin control of cell elongation. Nature 451: 480–484. 18216857 10.1038/nature06520

[nph70746-bib-0059] Mao J , Zhang Y‐C , Sang Y , Li Q‐H , Yang H‐Q . 2005. A role for Arabidopsis cryptochromes and COP1 in the regulation of stomatal opening. Proceedings of the National Academy of Sciences, USA 102: 12270–12275. 10.1073/pnas.0501011102 PMC1189306 16093319

[nph70746-bib-0060] Matthews JSA , Vialet‐Chabrand S , Lawson T . 2020. Role of blue and red light in stomatal dynamic behaviour. Journal of Experimental Botany 71: 2253–2269. 31872212 10.1093/jxb/erz563 PMC7134916

[nph70746-bib-0061] McNellis TW , Von Arnim AG , Araki T , Komeda Y , Miséra S , Deng XW . 1994. Genetic and molecular analysis of an allelic series of cop1 mutants suggests functional roles for the multiple protein domains. Plant Cell 6: 487–500. 8205001 10.1105/tpc.6.4.487 PMC160452

[nph70746-bib-0062] Merilo E , Laanemets K , Hu H , Xue S , Jakobson L , Tulva I , Gonzalez‐Guzman M , Rodriguez PL , Schroeder JI , Brosche M *et al*. 2013. PYR/RCAR receptors contribute to ozone‐, reduced air humidity‐, darkness‐, and CO2‐induced stomatal regulation. Plant Physiology 162: 1652–1668. 23703845 10.1104/pp.113.220608 PMC3707544

[nph70746-bib-0063] Michaud O , Krahmer J , Galbier F , Lagier M , Galvão VC , Ince YÇ , Trevisan M , Knerova J , Dickinson P , Hibberd JM *et al*. 2023. Abscisic acid modulates neighbor proximity‐induced leaf hyponasty in Arabidopsis. Plant Physiology 191: 542–557. 36135791 10.1093/plphys/kiac447 PMC9806605

[nph70746-bib-0064] Monte E , Alonso JM , Ecker JR , Zhang Y , Li X , Young J , Austin‐Phillips S , Quail PH . 2003. Isolation and characterization of *phyC* mutants in Arabidopsis reveals complex crosstalk between phytochrome signaling pathways. Plant Cell 15: 1962–1980. 12953104 10.1105/tpc.012971 PMC181324

[nph70746-bib-0065] Moriconi V , Binkert M , Costigliolo C , Sellaro R , Ulm R , Casal JJ . 2018. Perception of sunflecks by the UV‐B photoreceptor UV RESISTANCE LOCUS8. Plant Physiology 177: 75–81. 29530938 10.1104/pp.18.00048 PMC5933136

[nph70746-bib-0066] Munemasa S , Hauser F , Park J , Waadt R , Brandt B , Schroeder JI . 2015. Mechanisms of abscisic acid‐mediated control of stomatal aperture. Current Opinion in Plant Biology 28: 154–162. 26599955 10.1016/j.pbi.2015.10.010 PMC4679528

[nph70746-bib-0067] Murchie EH , Lawson T . 2013. Chlorophyll fluorescence analysis: a guide to good practice and understanding some new applications. Journal of Experimental Botany 64: 3983–3998. 23913954 10.1093/jxb/ert208

[nph70746-bib-0068] Musil CF , Wand SJE . 1993. Responses of sclerophyllous ericaceae to enhanced levels of ultraviolet‐B radiation. Environmental and Experimental Botany 33: 233–242.

[nph70746-bib-0069] Nogués S , Allen DJ , Morison JIL , Baker NR . 1999. Characterization of stomatal closure caused by ultraviolet‐B radiation. Plant Physiology 121: 489–496. 10517840 10.1104/pp.121.2.489 PMC59411

[nph70746-bib-0070] Ortiz‐Alcaide M , Llamas E , Gomez‐Cadenas A , Nagatani A , Martínez‐García JF , Rodríguez‐Concepción M . 2019. Chloroplasts modulate elongation responses to canopy shade by retrograde pathways involving HY5 and abscisic acid. Plant Cell 31: 384–398. 30705135 10.1105/tpc.18.00617 PMC6447015

[nph70746-bib-0071] Park S‐Y , Fung P , Nishimura N , Jensen DR , Fujii H , Zhao Y , Lumba S , Santiago J , Rodrigues A , Chow T‐FF *et al*. 2009. Abscisic acid inhibits type 2C protein phosphatases via the PYR/PYL family of START proteins. Science 324: 1068–1071. 19407142 10.1126/science.1173041 PMC2827199

[nph70746-bib-0072] Pei D , Hua D , Deng J , Wang Z , Song C , Wang Y , Wang Y , Qi J , Kollist H , Yang S *et al*. 2022. Phosphorylation of the plasma membrane H+‐ATPase AHA2 by BAK1 is required for ABA‐induced stomatal closure in Arabidopsis. Plant Cell 34: 2708–2729. 35404404 10.1093/plcell/koac106 PMC9252505

[nph70746-bib-0073] Pridgeon AJ , Hetherington AM . 2021. ABA signalling and metabolism are not essential for dark‐induced stomatal closure but affect response speed. Scientific Reports 11: 5751. 33707501 10.1038/s41598-021-84911-5 PMC7952387

[nph70746-bib-0074] Qi L , Liu S , Li C , Fu J , Jing Y , Cheng J , Li H , Zhang D , Wang X , Dong X *et al*. 2020. PHYTOCHROME‐INTERACTING FACTORS interact with the ABA receptors PYL8 and PYL9 to orchestrate ABA signaling in darkness. Molecular Plant 13: 414–430. 32059872 10.1016/j.molp.2020.02.001

[nph70746-bib-0075] R Core Team . 2021. R: a language and environment for statistical computing. Vienna, Austria: R Foundation for Statistical Computing.

[nph70746-bib-0076] Roth‐Bejerano N , Nejidat A , Itai C . 1990. Regulation of stomatal opening in epidermal strips of *Commelina communis* L. by phytochrome. Biochemie und Physiologie der Pflanzen 186: 375–379.

[nph70746-bib-0077] Rovira A , Veciana N , Basté‐Miquel A , Quevedo M , Locascio A , Yenush L , Toledo‐Ortiz G , Leivar P , Monte E . 2024. PIF transcriptional regulators are required for rhythmic stomatal movements. Nature Communications 15: 4540. 10.1038/s41467-024-48669-4 PMC11137129 38811542

[nph70746-bib-0078] Saito S , Hirai N , Matsumoto C , Ohigashi H , Ohta D , Sakata K , Mizutani M . 2004. Arabidopsis *CYP707A* s encode (+)‐Abscisic acid 8′‐hydroxylase, a key enzyme in the oxidative catabolism of abscisic acid. Plant Physiology 134: 1439–1449. 15064374 10.1104/pp.103.037614 PMC419820

[nph70746-bib-0079] Schindelin J , Arganda‐Carreras I , Frise E , Kaynig V , Longair M , Pietzsch T , Preibisch S , Rueden C , Saalfeld S , Schmid B *et al*. 2012. Fiji: an open‐source platform for biological‐image analysis. Nature Methods 9: 676–682. 22743772 10.1038/nmeth.2019 PMC3855844

[nph70746-bib-0080] Sharma A , Pridgeon AJ , Liu W , Segers F , Sharma B , Jenkins GI , Franklin KA . 2023. *ELONGATED HYPOCOTYL5* (*HY5*) and *HY5 HOMOLOGUE* (*HYH*) maintain shade avoidance suppression in UV‐B. The Plant Journal 115: 1394–1407. 37243898 10.1111/tpj.16328 PMC10953383

[nph70746-bib-0081] Sharma A , Sharma B , Hayes S , Kerner K , Hoecker U , Jenkins GI , Franklin KA . 2019. UVR8 disrupts stabilisation of PIF5 by COP1 to inhibit plant stem elongation in sunlight. Nature Communications 10: 4417. 10.1038/s41467-019-12369-1 PMC6764944 31562307

[nph70746-bib-0082] Sharma N . 2017. Leaf clearing protocol to observe stomata and other cells on leaf surface. Bio‐Protocol 7: e2538.

[nph70746-bib-0083] Shirley BW , Kubasek WL , Storz G , Bruggemann E , Koornneef M , Ausubel FM , Goodman HM . 1995. Analysis of *Arabidopsis* mutants deficient in flavonoid biosynthesis. The Plant Journal 8: 659–671. 8528278 10.1046/j.1365-313x.1995.08050659.x

[nph70746-bib-0084] Smit ME , Vatén A , Mair A , Northover CAM , Bergmann DC . 2023. Extensive embryonic patterning without cellular differentiation primes the plant epidermis for efficient post‐embryonic stomatal activities. Developmental Cell 58: 506–521. 36931268 10.1016/j.devcel.2023.02.014

[nph70746-bib-0085] Sutter J‐U , Sieben C , Hartel A , Eisenach C , Thiel G , Blatt MR . 2007. Abscisic acid triggers the endocytosis of the Arabidopsis KAT1 K+ channel and its recycling to the plasma membrane. Current Biology 17: 1396–1402. 17683934 10.1016/j.cub.2007.07.020

[nph70746-bib-0086] Takemiya A , Sugiyama N , Fujimoto H , Tsutsumi T , Yamauchi S , Hiyama A , Tada Y , Christie JM , Shimazaki K . 2013. Phosphorylation of BLUS1 kinase by phototropins is a primary step in stomatal opening. Nature Communications 4: 2094. 10.1038/ncomms3094 23811955

[nph70746-bib-0087] Talbott LD , Shmayevich IJ , Chung Y , Hammad JW , Zeiger E . 2003. Blue light and phytochrome‐mediated stomatal opening in the *npq1* and *phot1 phot2* mutants of Arabidopsis. Plant Physiology 133: 1522–1529. 14576287 10.1104/pp.103.029587 PMC300709

[nph70746-bib-0088] Talbott LD , Zhu J , Han SW , Zeiger E . 2002. Phytochrome and blue light‐mediated stomatal opening in the orchid, Paphiopedilum. Plant and Cell Physiology 43: 639–646. 12091717 10.1093/pcp/pcf075

[nph70746-bib-0089] Tavridou E , Pireyre M , Ulm R . 2020a. Degradation of the transcription factors PIF4 and PIF5 under UV‐B promotes UVR8‐mediated inhibition of hypocotyl growth in Arabidopsis. The Plant Journal 101: 507–517. 31571300 10.1111/tpj.14556 PMC7027837

[nph70746-bib-0090] Tavridou E , Schmid‐Siegert E , Fankhauser C , Ulm R . 2020b. UVR8‐mediated inhibition of shade avoidance involves HFR1 stabilization in Arabidopsis. PLoS Genetics 16: e1008797. 32392219 10.1371/journal.pgen.1008797 PMC7241853

[nph70746-bib-0091] Taylor G , Walter J , Kromdijk J . 2024. Illuminating stomatal responses to red light: establishing the role of *C* i‐dependent versus‐independent mechanisms in control of stomatal behaviour. Journal of Experimental Botany 75: 6810–6822. 38442206 10.1093/jxb/erae093 PMC11565200

[nph70746-bib-0092] Teramura AH . 1983. Effects of ultraviolet‐B radiation on the growth and yield of crop plants. Physiologia Plantarum 58: 415–427.

[nph70746-bib-0093] Tossi V , Lamattina L , Cassia R . 2009. An increase in the concentration of abscisic acid is critical for nitric oxide‐mediated plant adaptive responses to UV‐B irradiation. New Phytologist 181: 871–879. 19140950 10.1111/j.1469-8137.2008.02722.x

[nph70746-bib-0094] Tossi V , Lamattina L , Jenkins GI , Cassia RO . 2014. Ultraviolet‐B‐Induced stomatal closure in Arabidopsis is regulated by the UV RESISTANCE LOCUS8 photoreceptor in a nitric oxide‐dependent mechanism. Plant Physiology 164: 2220–2230. 24586043 10.1104/pp.113.231753 PMC3982774

[nph70746-bib-0095] Wang F‐F , Lian H‐L , Kang C‐Y , Yang H‐Q . 2010. Phytochrome B is involved in mediating red light‐induced stomatal opening in *Arabidopsis thaliana* . Molecular Plant 3: 246–259. 19965572 10.1093/mp/ssp097

[nph70746-bib-0096] Wickham H . 2016. ggplot2: elegant graphics for data analysis. New York, NY, USA: Springer‐Verlag.

[nph70746-bib-0097] Yoshida R , Hobo T , Ichimura K , Mizoguchi T , Takahashi F , Aronso J , Ecker JR , Shinozaki K . 2002. ABA‐activated SnRK2 protein kinase is required for dehydration stress signaling in Arabidopsis. Plant and Cell Physiology 43: 1473–1483. 12514244 10.1093/pcp/pcf188

[nph70746-bib-0098] Zhu M , Geng S , Chakravorty D , Guan Q , Chen S , Assmann SM . 2020. Metabolomics of red‐light‐induced stomatal opening in *Arabidopsis thaliana*: Coupling with abscisic acid and jasmonic acid metabolism. The Plant Journal 101: 1331–1348. 31677315 10.1111/tpj.14594

