# Supplementary material for: Integration of light quality signals regulates ABA abundance and stomatal movements during seedling establishment
Source: New Phytol. 2025 Nov 23;249(3):1253–67. doi: 10.1111/nph.70746 (PMC12780325; doi:10.1111/nph.70746)
Supplement: Supplementary file 1 — Fig. S1 Light spectra of the different treatments described in this study. Fig. S2 Cotyledons and rosette leaves differ in stomatal responses to light quality. Fig. S3 UV‐B‐mediated stomatal opening likely requires COP1, but not HY5/HYH. Fig. S4 Low dose UV‐B treatment overrides far‐red inhibition of opening in Chinese kale cotyledons. Fig. S5 UV‐B regulates PIF4 transcript abundance and induces stomatal opening in pif4/7 mutants but not uvr8‐6. Fig. S6 UV‐B‐mediated increases in stomatal aperture do not result from small elevations in cotyledon temperature. Fig. S7 uvr8 and pif4 mutants show no significant differences in cotyledon stomatal density. Fig. S8 FR and UV‐B supplementation affect the transcript levels of genes involved in ABA metabolism. Fig. S9 FR and UV‐B supplementation affect the transcript levels of genes involved in ABA signalling. Fig. S10 QPCR of candidate low R : FR and low dose UV‐B response genes. Fig. S11 Low dose UV‐B supplementation has only minor effects on maximum photosystem II efficiency. [file NPH-249-1253-s001.docx]

**New Phytologist Supporting Information**
**Article title:** Integration of light quality signals regulates ABA abundance and stomatal movements during seedling establishment
**Authors:** Mathilda Gustavsson, Lionel Hill, Keara A. Franklin, Ashley J. Pridgeon
**Article acceptance date:** 24 October 2025

# Supporting Information


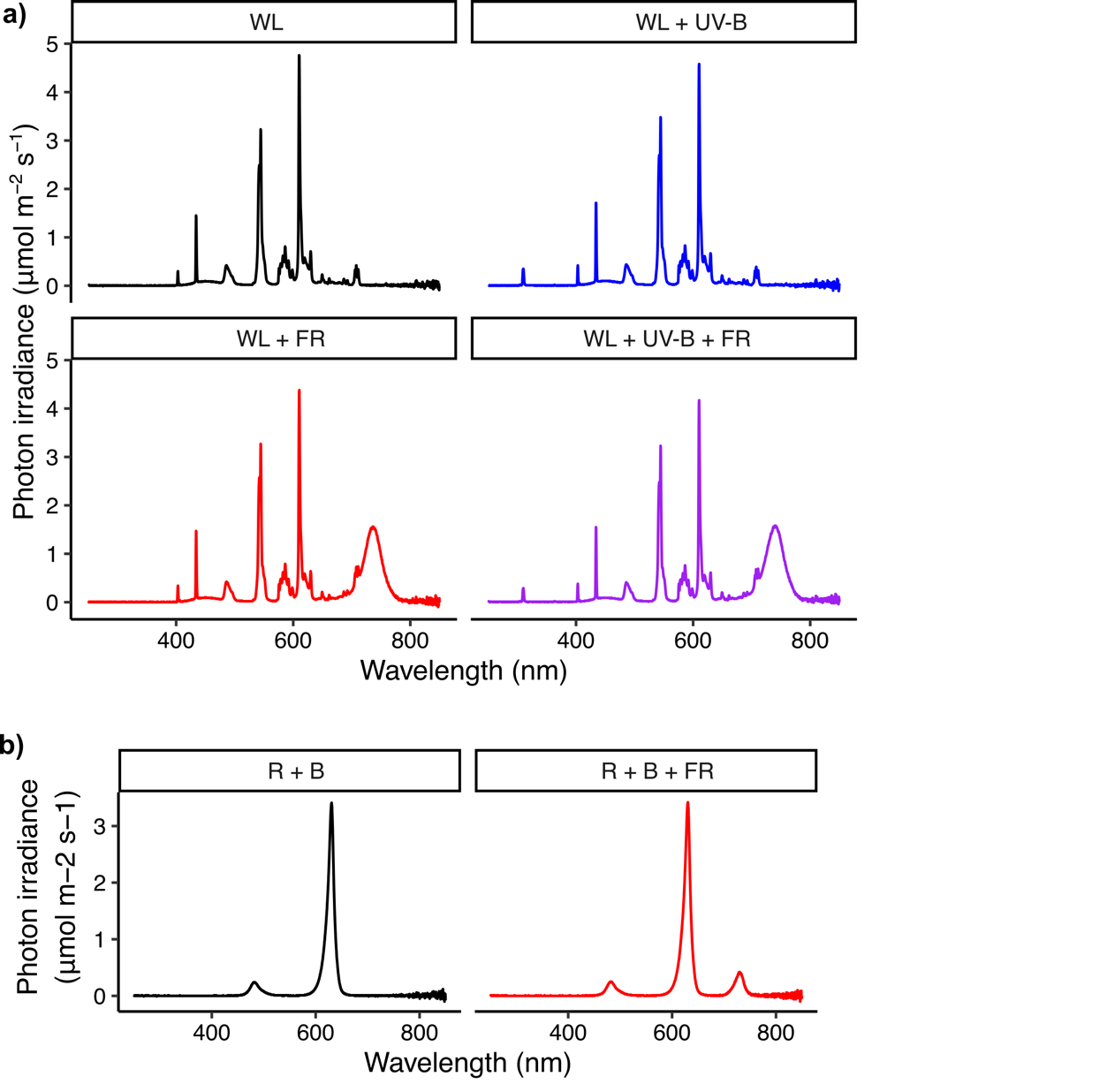


## Fig. S1 – Light treatment spectra

Light spectra of the different treatments described in this study. **a)** Treatments include ~75 µmolm^-2^s^-1^ white light (light between 400-700 nm). UV-B treatments involved the addition of 1 µmolm^-2^s^-1^ of UV-B light provided by a Philips TL100W/01 narrow band tube light. Far red (FR) treatments involved the addition of FR LEDs with a maximum emission peak at 735 nm. **b)** Red and blue (R +B) ± FR light treatments used for gas exchange measurements. Treatments involved 67 µmolm^-2^s^-1^ red light, 8 µmolm^-2^s^-1^ blue light (R + B), and when applicable 10 µmolm^-2^s^-1^ FR light. Light is provided by the Licor 6800 multiphase flash fluorometer.


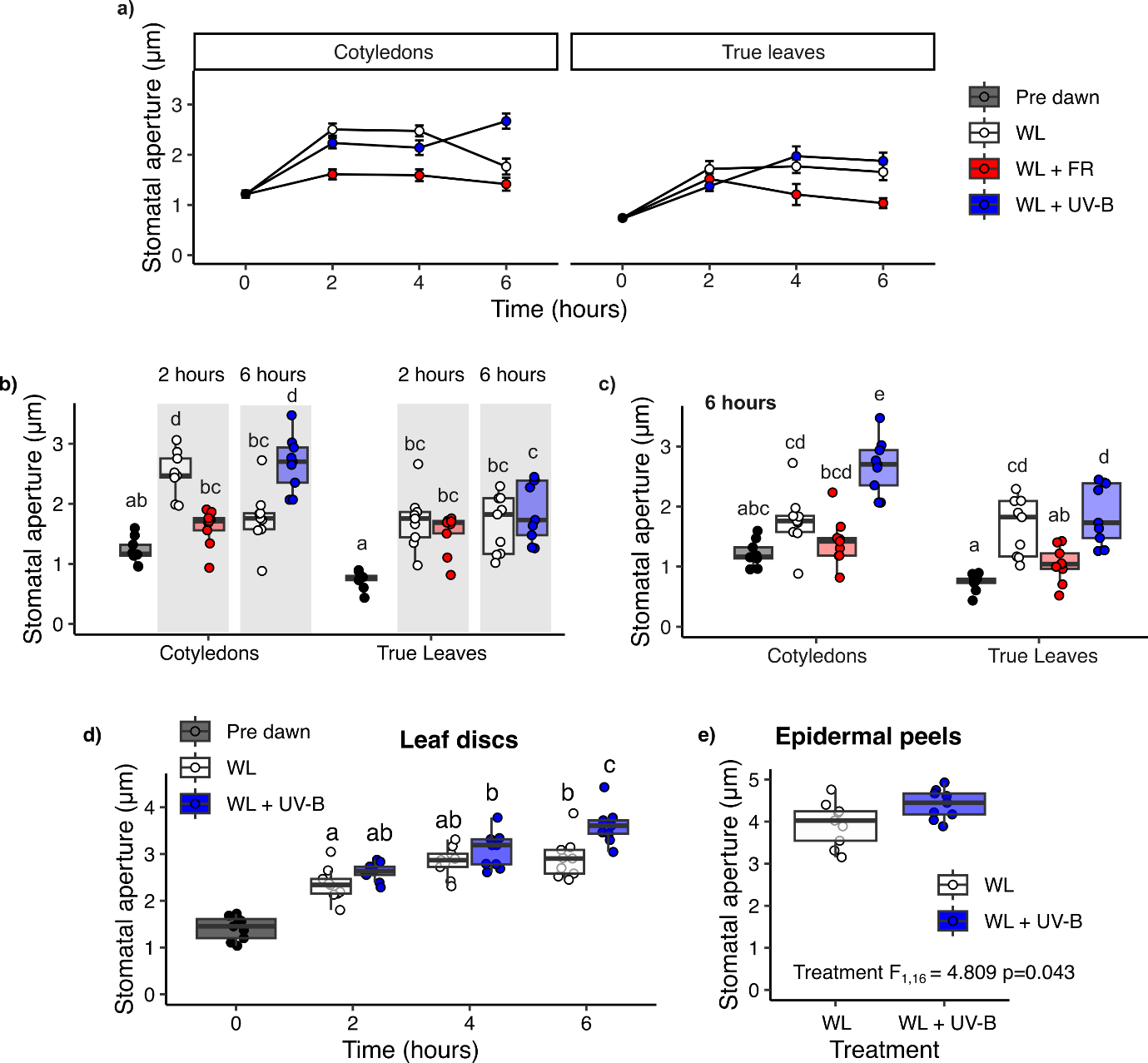


## Fig. S2 – Cotyledons and rosette leaves differ in stomatal responses to light quality

**a)** Stomatal apertures from 7-day-old seedling cotyledons and 21-day-old true leaves were measured in white light (WL), WL ± far red (FR), and WL ± UV-B conditions over a 6 hour time course. Selected timepoints from **a)** are shown in **b)** 2h WL ± FR and 6h WL ± UV-B and **c)** all light treatments at 6 hours. Stomatal apertures from **d)** leaf discs and **e)** epidermal peels of 4-5 week-old Col-0 plants. Apertures were measured **d)** pre-dawn and over a 6 h time course (2, 4, and 6 h) of WL ± UV-B treatment. In **e)** apertures were measured following 6 hours of WL ± UV-B treatment. For **a)** data is represented as mean ± standard error (s.e.). For **b-e)**, data are presented as boxplots showing the median and interquartile range (IQR) of each group. The upper and lower whiskers represent data within 1.5 * IQR. Each individual plant’s mean stomatal aperture calculated from 10-12 stomatal measurements is represented as a point on the plot. For all genotype treatment combinations, n = 9 plants over 3 independent experiments. Data in **b-d)** were analysed using 2-way ANOVA followed by Tukey Multiple Comparisons tests. Data in **e)** was analysed using a Students T test. Letters indicate significance at p < 0.05.


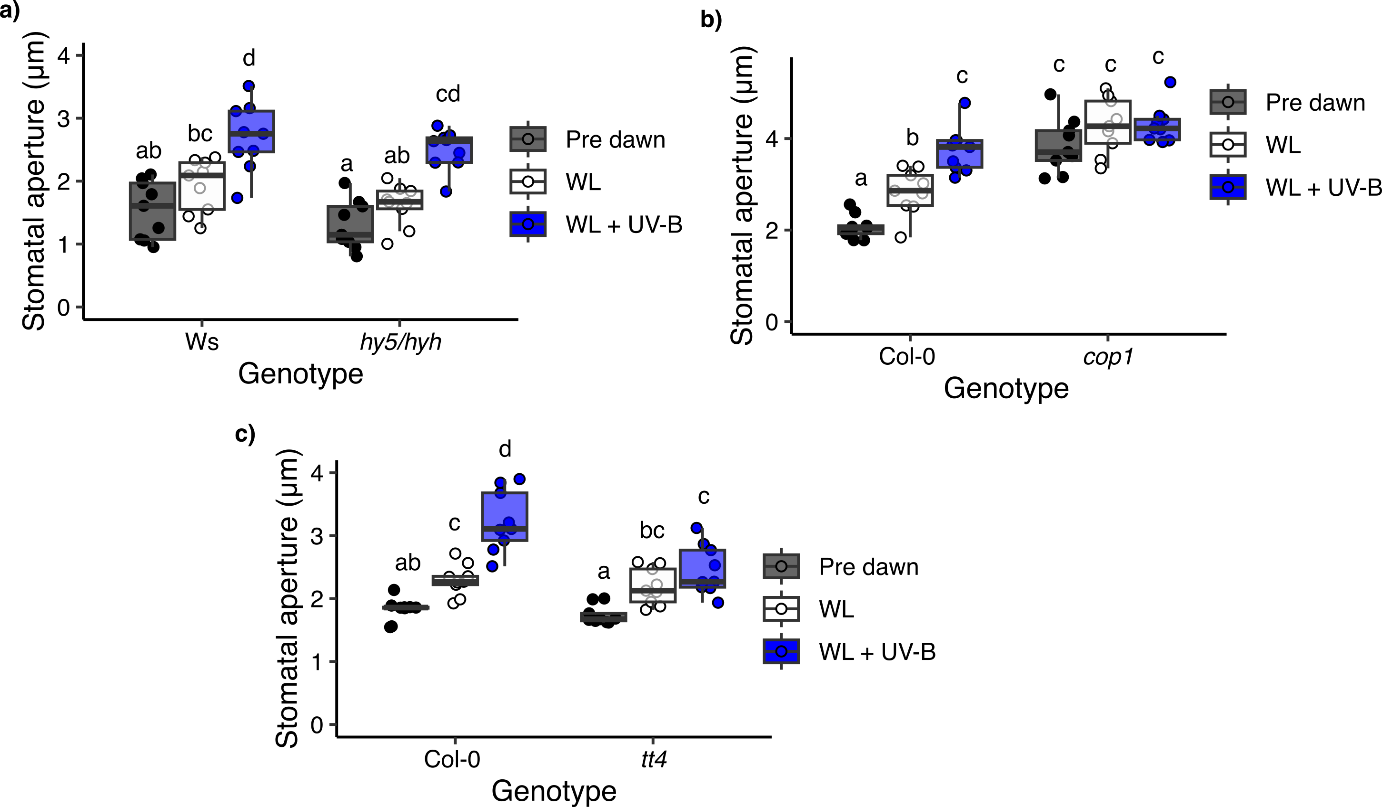


## Fig. S3 – UV-B-mediated stomatal opening likely requires COP1, but not HY5/HYH

The stomatal apertures of **a)** *hy5hyh*, **b)** *cop1*, and **c)** *tt4* mutants following 6 h of white light (WL) ± UV-B treatment. Stomatal apertures were also measured prior to dawn (Pre dawn). Data are presented as boxplots showing the median and interquartile range of each group. The upper and lower whiskers represent data within 1.5 * IQR. Each individual plant’s mean stomatal aperture is represented as a point on the plot. For all genotype and treatment combinations, n = 9 seedlings over 3 independent experiments. Each mean seedling stomatal aperture was calculated from 10 stomatal measurements. Data were analysed using a 2-way ANOVA, followed by Tukey multiple comparison test. In **b)** *cop1* was grown in parallel with *uvr8-6* (**Fig. 1C**) so both plots use the same Col-0 control data.


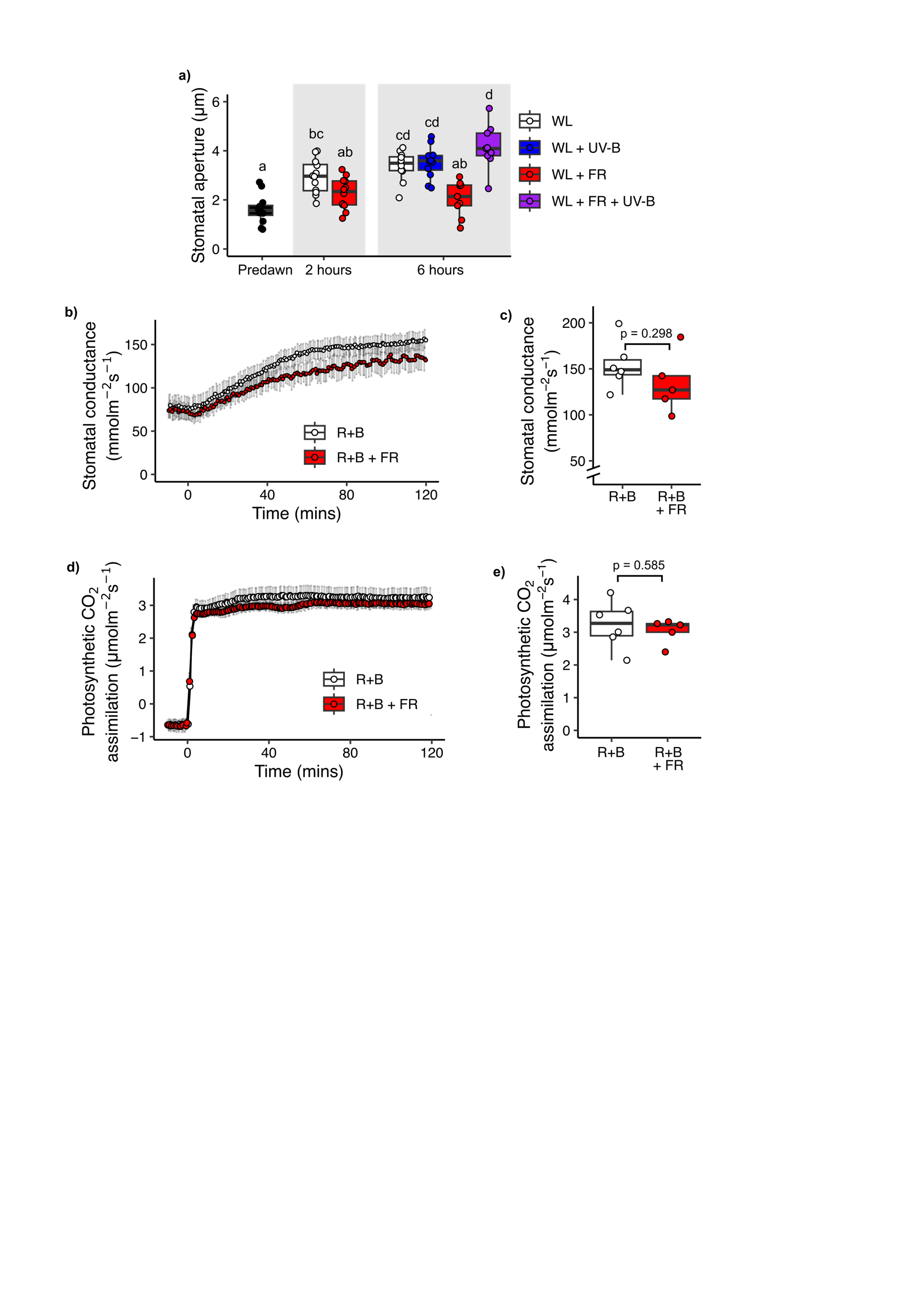


## Fig. S4 – Low dose UV-B treatment overrides far red inhibition of opening in Chinese kale cotyledons

**a)** Stomatal aperture width of 8-day-old Chinese kale cotyledons treated with white light (WL) ± far red (FR) ± UV-B for a maximum of 6 hours. Gas exchange parameters were measured over a 2-hour time-course for 14-18 day old Chinese kale cotyledons in response to red and blue (R+B) light ± FR. **b)** Stomatal conductance and **d)** photosynthetic CO_2_ assimilation are presented over the time-course. The average **c)** Stomatal conductance and **e)** photosynthetic CO_2_ assimilation between the 110 - 120 min time-points are presented for each seedling. For **a)** data are presented as boxplots showing the median and interquartile range (IQR) of each group. The upper and lower whiskers represent data within 1.5 * IQR. All data are shown as points overlayed on-top of the boxplots n = 9-12 plants over 3-4 independent experiments. For **b)** and **d)** data are presented as mean ± standard error (s.e.). For **c)** and **e)** data are presented as boxplots and n = 5-6. The data in **a)** was analysed using a 1-way ANOVA, and in **c)** and **e)** using a students t-test.


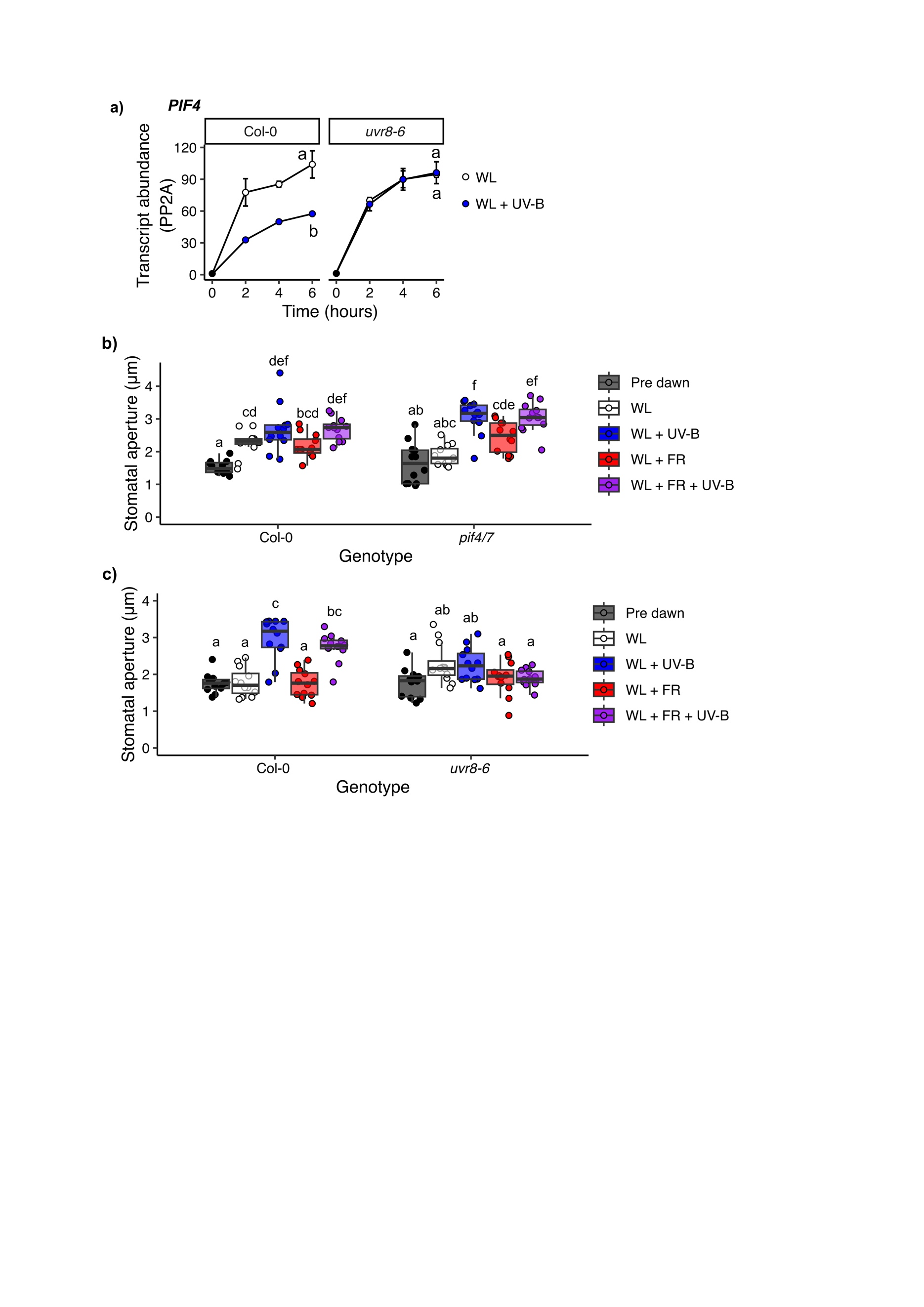


## Fig. S5 – UV-B regulates PIF4 transcript abundance and induces stomatal opening in pif4/7 mutants but not uvr8-6

**a)** *PIF4* transcript abundance measure over a 6 hour time course under white light (WL) ± UV-B in Col-0 and *uvr8-6* seedlings. n = 3 samples over 3 independent experiments. Data in **a)** are presented as mean ± standard error (s.e.). Data from 6 hours were analysed using 2-way ANOVA with Tukey multiple comparison tests. The stomatal apertures of **b)** *pif4/7* and **c)** *uvr8-6* mutants following 6 h of WL ± far red (FR) ± UV-B. Stomatal apertures were also measure prior to dawn (Pre dawn). Data in **b)** and **c)** are presented as boxplots showing the median and interquartile range (IQR) of each group. The upper and lower whiskers represent data within 1.5 * IQR. Each individual plant’s mean stomatal aperture is represented as a point on the plot. For all genotype and treatment combinations, n = 12 seedlings over 4 independent experiments. Each mean seedling stomatal aperture was calculated from 12 stomatal measurements. Data were analysed using a 2-way ANOVA, followed by Tukey multiple comparison tests.


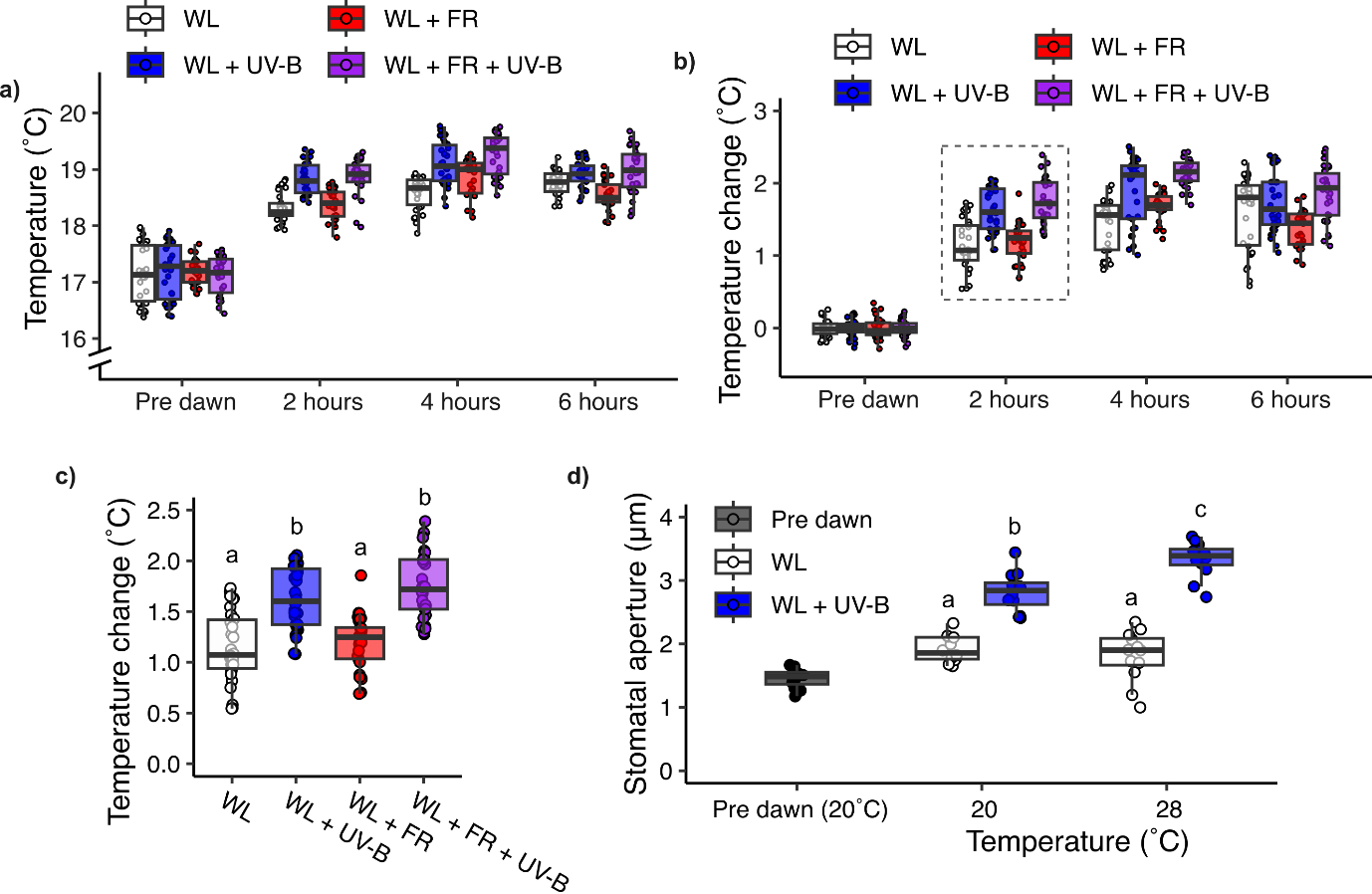


## Fig. S6 – UV-B-mediated increases in stomatal aperture do not result from small elevations in cotyledon temperature

**a-c)** Cotyledon temperature measurements from 7-day-old Col-0 seedlings treated with white light (WL) ± far red (FR) and/or UV-B from dawn. **a)** Absolute temperature measurements and **b)** Temperature change (relative to pre dawn values) over a 6 hour time-course. **c)** Temperature change at 2 hours (indicated by dashed box in **b**). **d)** Mean stomatal apertures of 7 day old Col-0 seedlings treated with WL ± UV-B at 20˚C and 28˚C for 6 hours following dawn. All data presented as points overlayed on top of boxplots showing the median and interquartile range (IQR) of each group. The upper and lower whiskers represent data within 1.5 * IQR. For **a-c)** all treatment combinations n = 10 seedlings over 3 independent experiments. For **d)** each mean stomatal aperture was calculated from 8-12 stomatal measurements. A total of n = 12 seedlings were analysed at each temperature treatment combination over 3 independent experiments. Data in **c)** was analysed using a 1-way ANOVA looking at the effect of light treatment, and in **d)** using a 2-way ANOVA looking at the effect of temperature and light treatment (pre-dawn values were not included in statistical analysis) followed by Tukey multiple comparison tests. Letters indicate significance at p < 0.05.


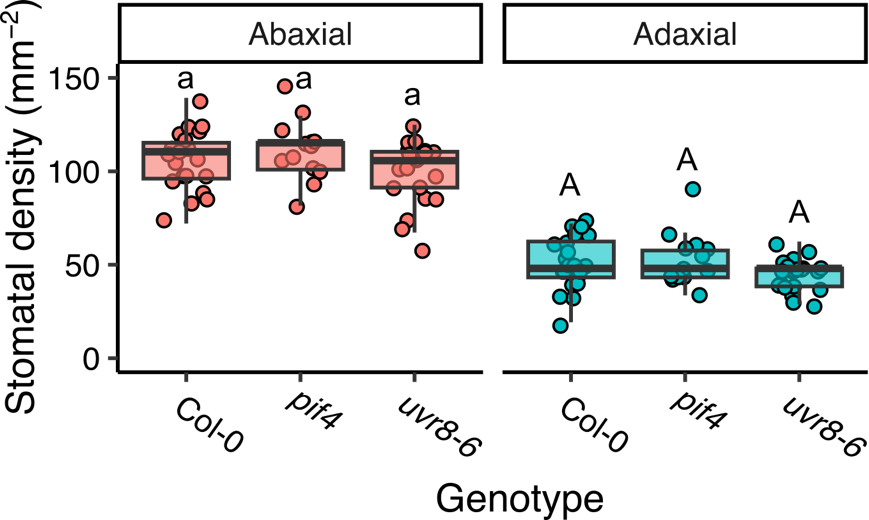


## Fig. S7 – uvr8 and pif4 mutants show no significant differences in cotyledon stomatal density

The abaxial and adaxial stomatal densities of 7-day-old Col-0, *pif4-101*, and *uvr8-6*. All data is presented as points overlayed on top of boxplots showing the median and interquartile range (IQR) of each group. The upper and lower whiskers represent data within 1.5 * IQR. n = 13-21 over 2 independent experiments. The abaxial and adaxial data in were separately analysed using a 1-way ANOVA. Multiple comparisons were performed using a post hoc Tukey test. Letters denote significance at p < 0.05.


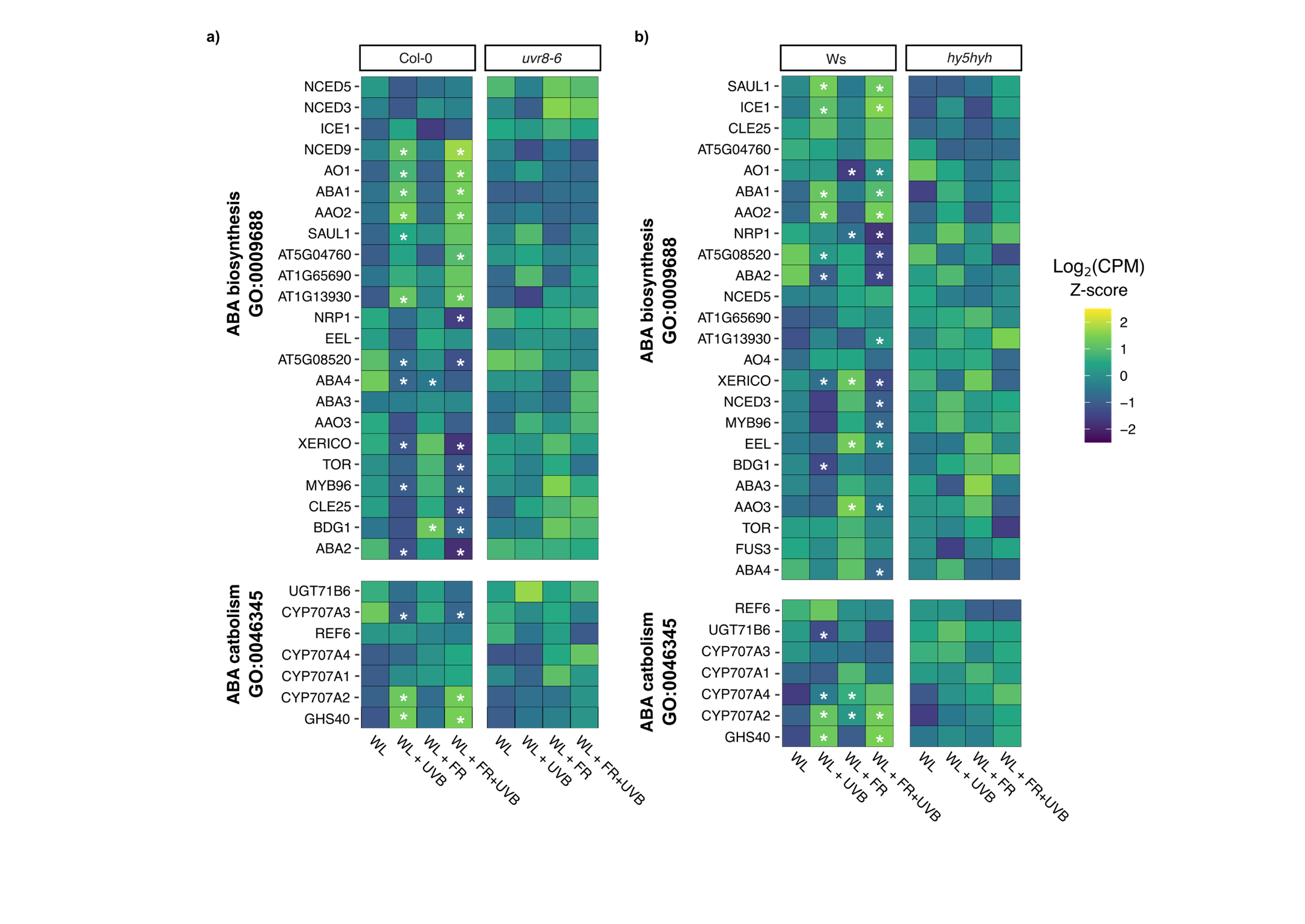


## Fig. S8 – FR and UV-B supplementation affect the transcript levels of genes involved in ABA metabolism

Analysis of transcripts tagged with GO terms related to ABA metabolism (biosynthesis and catabolism) from **a)** Tavridou et al. 2020b and **b)** Sharma et al. 2023. Transcript levels are presented as the z-score of the log_2_(counts per million/CPM) for each gene. Gene tiles in the white light (WL) + UV-B and WL + far red (FR) columns marked with asterixes are considered differentially expressed compared to WL conditions. Gene tiles marked with asterixes in the WL+FR+UV-B column are considered differentially expressed compared to WL+FR conditions.


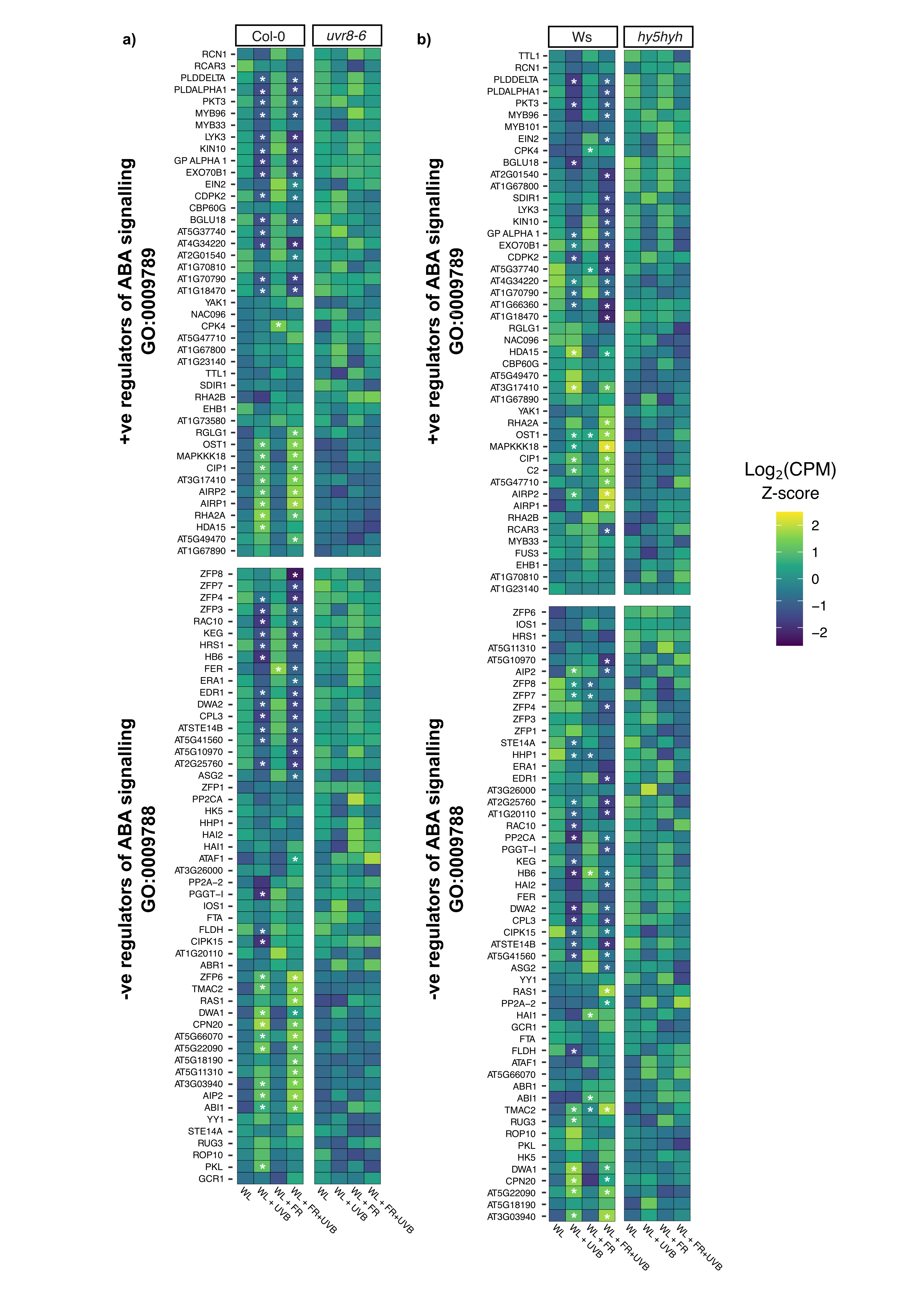


## Fig. S9 - FR and UV-B supplementation affect the transcript levels of genes involved in ABA signalling

Analysis of transcripts tagged with GO terms related to ABA signalling from **a)** Tavridou et al. 2020b and **b)** Sharma et al. 2023. Transcript levels are presented as the z-score of the log_2_(counts per million/CPM) for each gene. Gene tiles within the wild-type groups in the white light (WL) + UV-B and WL + far red (FR) columns marked with asterixes are considered differentially expressed compared to WL conditions. Gene tiles marked within the wild-type groups with asterixes in the WL + FR + UV-B column are considered differentially expressed compared to WL + FR conditions.


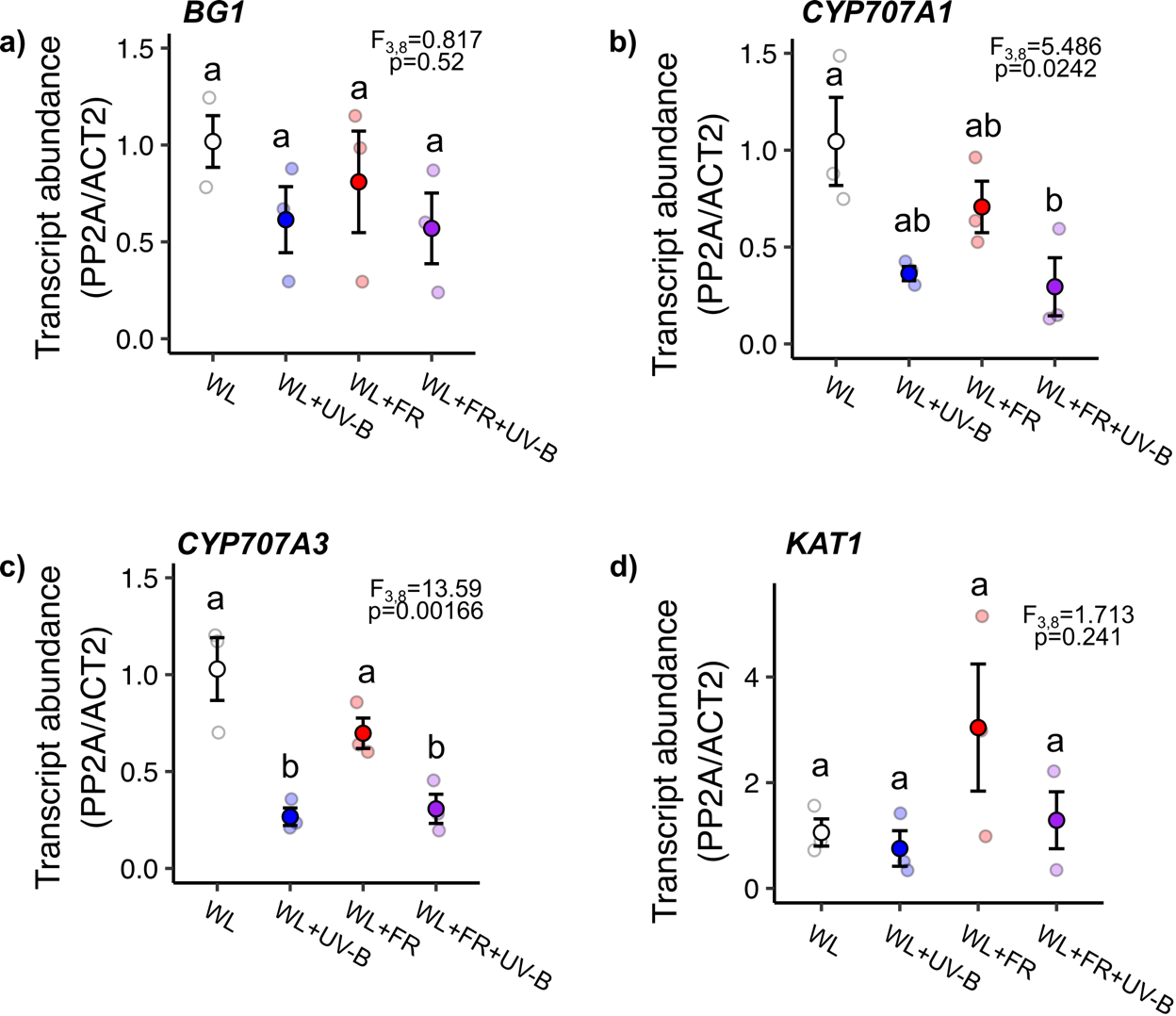


## Fig. S10 – QPCR of candidate low R:FR and low dose UV-B response genes

**a)** *BG1*, **b)** *CYP707A1*, **c)** *CYP707A3,* and **d)** *KAT1* transcript abundance was measured in the aerial tissue of 7-day-old Arabidopsis seedlings treated with white light (WL) ± far red (FR) ± UV-B light for 6 hours. For all data n = 3 samples over 3 independent experiments. Data were analysed using 1-way ANOVA with post-hoc Tukey multiple comparison tests.


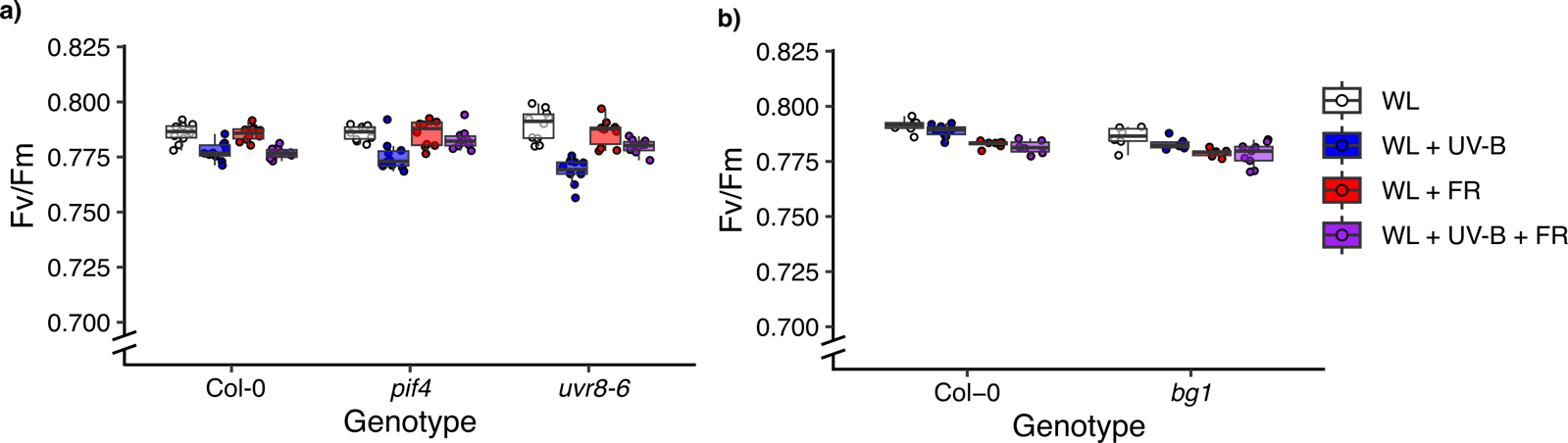


## Fig. S11 – Low dose UV-B supplementation has only minor effects on maximum photosystem II efficiency

**a-b)** F_V_/F_M_ measurements for 7-day-old seedlings following 6 hours of white light (WL) ± far red (FR) ± UV-B treatment and 30 mins of dark adaption. Col-0 with **a)** *pif4-101* and *uvr8-6*, and **b)** *bg1* mutants are presented. Data in **a-b)** is one repeat representative of 3 independent experiments. Each experiment consists of n = 10 seedling F_V_/F_M_ measurements.

# References

De Lucas M, Davière J-M, Rodríguez-Falcón M, Pontin M, Iglesias-Pedraz JM, Lorrain S, Fankhauser C, Blázquez MA, Titarenko E, Prat S. 2008. A molecular framework for light and gibberellin control of cell elongation. Nature 451: 480–484.

Favory J-J, Stec A, Gruber H, Rizzini L, Oravecz A, Funk M, Albert A, Cloix C, Jenkins GI, Oakeley EJ, et al. 2009. Interaction of COP1 and UVR8 regulates UV-B-induced photomorphogenesis and stress acclimation in Arabidopsis. The EMBO Journal 28: 591–601.

Franklin KA, Davis SJ, Stoddart WM, Vierstra RD, Whitelam GC. 2003. Mutant Analyses Define Multiple Roles for Phytochrome C in Arabidopsis Photomorphogenesis. The Plant Cell 15: 1981–1989.

Frey A, Effroy D, Lefebvre V, Seo M, Perreau F, Berger A, Sechet J, To A, North HM, Marion‐Poll A. 2012. Epoxycarotenoid cleavage by NCED5 fine‐tunes ABA accumulation and affects seed dormancy and drought tolerance with other NCED family members. The Plant Journal 70: 501–512.

Holm M, Ma L-G, Qu L-J, Deng X-W. 2002. Two interacting bZIP proteins are direct targets of COP1-mediated control of light-dependent gene expression in Arabidopsis. Genes & Development 16: 1247–1259.

Kagawa T, Sakai T, Suetsugu N, Oikawa K, Ishiguro S, Kato T, Tabata S, Okada K, Wada M. 2001. Arabidopsis NPL1: A Phototropin Homolog Controlling the Chloroplast High-Light Avoidance Response. Science 291: 2138–2141.

Lee KH, Piao HL, Kim H-Y, Choi SM, Jiang F, Hartung W, Hwang I, Kwak JM, Lee I-J, Hwang I. 2006. Activation of Glucosidase via Stress-Induced Polymerization Rapidly Increases Active Pools of Abscisic Acid. Cell 126: 1109–1120.

Leivar P, Monte E, Al-Sady B, Carle C, Storer A, Alonso JM, Ecker JR, Quail PH. 2008. The Arabidopsis phytochrome-interacting factor PIF7, together with PIF3 and PIF4, regulates responses to prolonged red light by modulating phyB levels. The Plant Cell 20: 337–352.

McNellis TW, Von Arnim AG, Araki T, Komeda Y, Miséra S, Deng XW. 1994. Genetic and molecular analysis of an allelic series of cop1 mutants suggests functional roles for the multiple protein domains. The Plant Cell 6: 487–500.

Park S-Y, Fung P, Nishimura N, Jensen DR, Fujii H, Zhao Y, Lumba S, Santiago J, Rodrigues A, Chow TF, et al. 2009. Abscisic Acid Inhibits Type 2C Protein Phosphatases via the PYR/PYL Family of START Proteins. Science 324: 1068–1071.

Sharma A, Pridgeon AJ, Liu W, Segers F, Sharma B, Jenkins GI, Franklin KA. 2023. ELONGATED HYPOCOTYL5 (HY5) and HY5 HOMOLOGUE (HYH) maintain shade avoidance suppression in UV‐B. The Plant Journal 115: 1394–1407.

Shirley BW, Kubasek WL, Storz G, Bruggemann E, Koornneef M, Ausubel FM, Goodman HM. 1995. Analysis of Arabidopsis mutants deficient in flavonoid biosynthesis. The Plant Journal 8: 659–671.

Takemiya A, Sugiyama N, Fujimoto H, Tsutsumi T, Yamauchi S, Hiyama A, Tada Y, Christie JM, Shimazaki K. 2013. Phosphorylation of BLUS1 kinase by phototropins is a primary step in stomatal opening. Nature Communications 4: 2094.

Tavridou E, Schmid-Siegert E, Fankhauser C, Ulm R. 2020. UVR8-mediated inhibition of shade avoidance involves HFR1 stabilization in Arabidopsis (O Mittelsten Scheid, Ed.). PLOS Genetics 16: e1008797.

Yoshida R, Hobo T, Ichimura K, Mizoguchi T, Takahashi F, Aronso J, Ecker JR, Shinozaki K. 2002. ABA-Activated SnRK2 Protein Kinase is Required for Dehydration Stress Signaling in Arabidopsis. Plant and Cell Physiology 43: 1473–1483.
